# Application of multiple omics and network projection analyses to drug repositioning for pathogenic mosquito-borne viruses

**DOI:** 10.1038/s41598-021-89171-x

**Published:** 2021-05-12

**Authors:** Takayuki Amemiya, Katsuhisa Horimoto, Kazuhiko Fukui

**Affiliations:** 1 Cellular and Molecular Biotechnology Research Institute, National Institute of Advanced Industrial Science and Technology (AIST), Tokyo, 135-0064 Japan; 2 Molecular Profiling Research Center for Drug Discovery (Molprof), National Institute of Advanced Industrial Science and Technology (AIST), Tokyo, 135-0064 Japan

**Keywords:** Viral infection, Predictive medicine, Proteome informatics, Biochemical networks

## Abstract

Pathogenic mosquito-borne viruses are a serious public health issue in tropical and subtropical regions and are increasingly becoming a problem in other climate zones. Drug repositioning is a rapid, pharmaco-economic approach that can be used to identify compounds that target these neglected tropical diseases. We have applied a computational drug repositioning method to five mosquito-borne viral infections: dengue virus (DENV), zika virus (ZIKV), West Nile virus (WNV), Japanese encephalitis virus (JEV) and Chikungunya virus (CHIV). We identified signature molecules and pathways for each virus infection based on omics analyses, and determined 77 drug candidates and 146 proteins for those diseases by using a filtering method. Based on the omics analyses, we analyzed the relationship among drugs, target proteins and the five viruses by projecting the signature molecules onto a human protein–protein interaction network. We have classified the drug candidates according to the degree of target proteins in the protein–protein interaction network for the five infectious diseases.

## Introduction

Pathogenic viruses cause diseases by infecting and replicating in human cells^1^, and the many varieties of mosquito pathogenic viruses combined cause more human suffering than any other organism. Mosquito-borne infections are widespread with over 390 million people infected annually^2^. Compared with other infectious diseases such as Ebola, Marburg, Crimea Congo and Lassa, the mortality rate of mosquito-borne infectious diseases is relatively low. Nonetheless, more than 50 billion people have died from mosquito-borne diseases^3^, which include dengue virus (DENV), Zika virus (ZIKV), malaria, West Nile virus (WNV), Chikungunya virus (CHIV), yellow fever virus (YFV) and the Japanese encephalitis virus (JEV). Infectious diseases such as DENV, ZIKV, WNV, YFV and JEV belong to the single genus flavivirus, and cause symptoms ranging from mild fever to more severe symptoms including encephalitis and hemorrhagic fever^4^. CHIV belongs to an arthritogenic alphavirus and causes high fever and severe joint pain. Mosquito-borne diseases may be spreading into regions of the world that have not recorded cases of such diseases because of the spread of mosquito habitats and the effects of global warming, especially changes in temperature and rainfall^5^. Current trends indicate that mosquito-borne diseases will affect greater numbers of people and the number of patients with serious symptoms will increase dramatically^6,7^. Thus, a better understanding of mosquito-borne infections is important for predicting future outbreaks of these pathogens. Mosquito-borne diseases are mainly treated symptomatically with only a few effective drugs available. Thus, treatment of these primarily tropical diseases has been neglected^8^ and development of new therapeutic drugs has not advanced because of a lack of investment into research. Drug repositioning/repurposing has been a recent cost-effective approach to find compounds that show limited side effects and are suitable to treat these rare and/or neglected tropical diseases^9^. Such an approach is cost effective for treating diseases with a small number of patients (rare diseases) and those populations living predominantly in poverty (e.g., tropics), where no profitable market for the development and sales of new drugs exist for pharmaceutical companies.

We have previously developed a multi-omics drug repositioning method to use the large-scale intergradation of experimentally measured data aimed at data-driven science^10^. In our computational approach, repositioning drug candidates for dengue hemorrhagic fever (DHF) syndrome that are used to treat other infectious diseases caused by flaviviruses was successful. For mosquito-borne infectious diseases, many research efforts have measured omics data and made data public using the rapid development of experimental technologies. Our multi-omics drug repositioning approach for identifying drug candidates to treat infectious diseases is very compatible and enables a comprehensive view of biological processes and biological networks that cross different molecular layers. We have surveyed a complete set of experimental data in three omics layers of open, accessible and high-quality data in pathogenetic mosquito-borne viruses. DENV, ZIKV, WNV and JEV from flaviviruses and CHIV from togaviruses were selected. YFV is also an arbovirus of the flavivirus genus. However, YFV was not analyzed because expression data for infected (healthy) versus non-infected (control) samples were not available at the Gene Expression Omnibus (GEO), and a literature search provided no interactome data. In this report, we identified signature genes, target proteins and target pathways for DENV, ZIKV, WNV, CHIV and JEV by analyzing published transcriptome, proteome and protein–protein interaction data. We determined approved drug candidates against each virus by integrating the results of omics analyses and applying our published filtering method^10^. The drug candidates were grouped by clinical symptoms: hemorrhagic syndrome (DENV), neurological complications (ZIKV, WNV and JEV) and inflammatory arthritis (CHIV). Based on the results obtained from multi-omics analyses, we aimed to classify the drugs by investigating communication routes of the five diseases using a network analysis approach. The analysis projects the signature molecules onto human protein–protein interactions to provide an overview of the shared relationships among drugs, diseases and signature molecules. Here, we first present drug candidates for rare and/or neglected tropical diseases, especially mosquito-borne diseases, by analyzing molecular profiling experimentally obtained from multiple omics, and then apply a network-based analysis to select drug candidates by exploring the relevance between the drug and the disease through protein interactions.

## Materials and methods

### Approach overview

**Figure 1:**
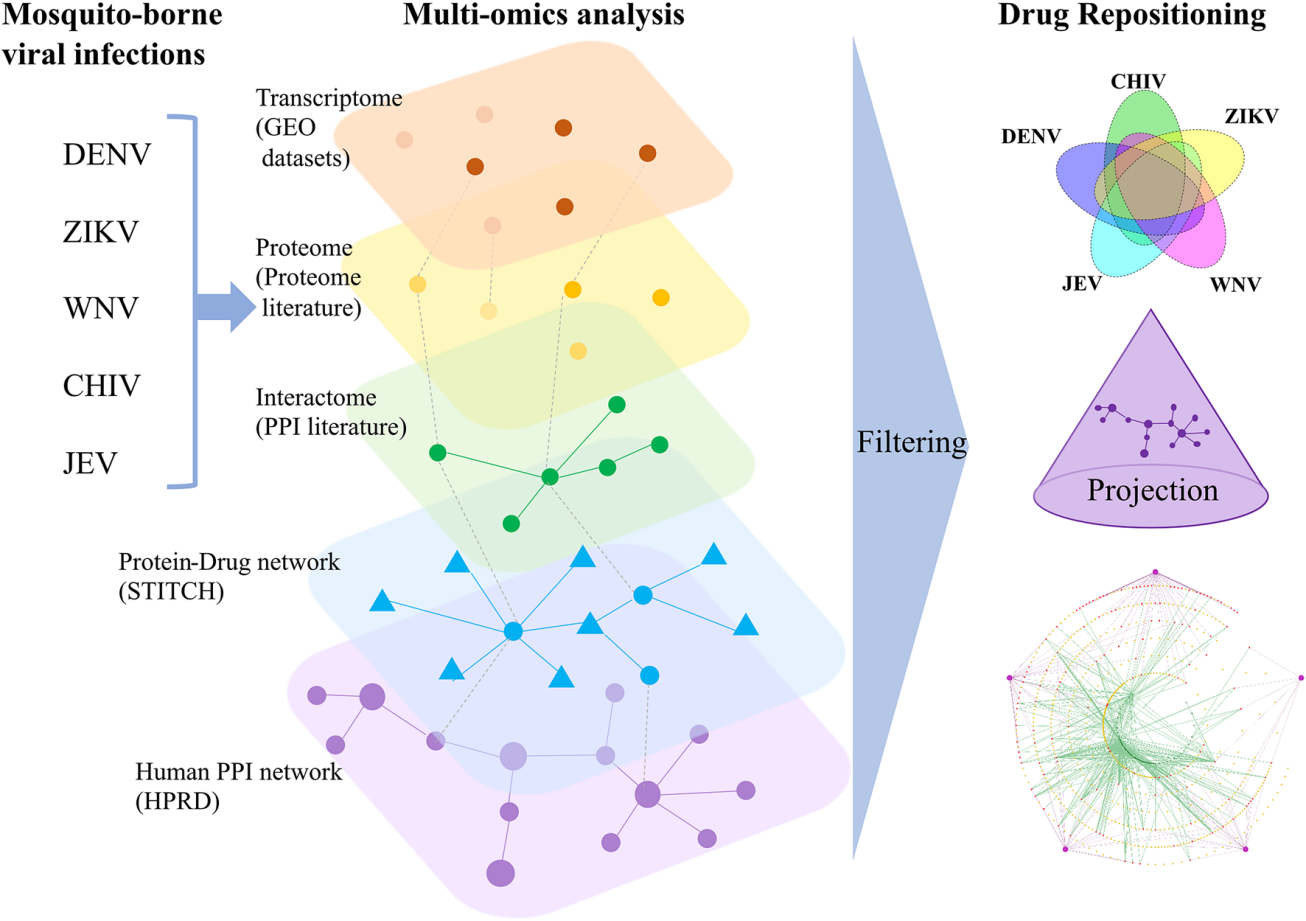
Schematic showing the process of drug repositioning against the five viruses (DENV, ZIKV, WNV, CHIV and JEV). Signature genes, signature proteins and interactions between human and viral proteins were selected from GEO datasets, review of the proteome literature and review of the human–viral PPI literature, respectively. To find drug candidates, a connectivity map (CMap) was generated for signature genes based on GEO data. STITCH was used for selecting drugs that interacted with the identified signature proteins from the proteome and human proteins in human–viral PPIs. Using our filtering method, we identified drug candidates for the five viral infections based on multiple omics analyses. Those disease-related proteins that control the onset and progression of the five viral infectious diseases were projected onto the protein–protein interaction network of the HPRD to identify drugs that are effective against the five diseases.

Figure 1 outlines the approach used in this study. Omics analysis of DENV, ZIKV, WNV, CHIV and JEV was performed using the following methods. Transcriptome analysis was performed by extracting gene expression data of patients and viral infected cells from GEO datasets^11^ to obtain signature genes. Signature genes are a set of genes that have an altered expression pattern between normal and infected patients, or uninfected and infected cells. Using those genes, Gene Set Enrichment Analysis (GSEA)^12^ was performed to detect disease-specific pathways related to each of the five infections. Literature searches were performed to find studies that provide proteomic data. Identified signature proteins were defined by protein expression patterns that differed between uninfected and infected cells. Literature surveys were also performed to find protein–protein interactions (PPIs) between human and viral proteins. A network of PPIs was obtained for interactome analysis to identify human proteins that interact with virus proteins associated with DENV, ZIKV, WNV, CHIV and JEV. Integrating the transcriptome, protein and interactome analyses enabled identification of common signature molecules.

To find drug candidates by the drug repositioning method, connectivity map (CMap) and STITCH were used for the signature genes derived from GEO data analysis and for chemicals that interacted with the identified signature proteins from the proteomics analysis and identified PPIs between human and viral proteins, respectively. Using our filtering method, drug candidates for the five viral infections were identified based on multiple omics analyses. Those disease-related proteins that could be used to control the onset and progression of the five infectious diseases were projected onto the protein–protein interaction network of the Human Protein Reference Database^13^, and the drugs were classified according to the degree of target proteins in PPIs for the five diseases.

### Datasets for transcriptomic analysis

The 16 microarray gene expression datasets related to DENV, ZIKV, WNV, CHIV and JEV infected cells were selected from the GEO database^14^ (Supplementary Table S1). To compare the effective gene signatures for each infectious disease in microarray differential expression analysis, we selected signature genes with fold-change (FC) values, which are more reproducible than those selected by statistical significance and could minimize variation due to sample differences^15^. The rank-based approach obtained from FC values for different sources is more comparable and leads to higher prediction accuracy than using expression values^16^. For the single ZIKV, CHIV and JEV datasets, we analyzed 1000 signature genes: the 500 most upregulated and the 500 most downregulated FC genes. For DENV and WNV with multiple datasets available, each fold change value was calculated as a mean of all experiments. The 500 most upregulated and 500 most downregulated FC genes determined by examining the mean FC values were then analyzed.

### Datasets for proteomic analyses

PubMed (https://www.ncbi.nlm.nih.gov/pubmed/) and Google scholar (https://scholar.google.com/) were used for searching literature that gave proteomic analyses of cells affected by the five viruses. We identified relevant studies using the keywords “proteomics analysis” and “proteome”. Proteins for proteomic analysis were extracted by reading all articles identified from the keyword search results. All proteomic datasets are presented in Supplementary Table S2. The number of unique identified signature proteins were 389 for DENV, 453 for ZIKV, 627 for WNV, 517 for CHIV and 183 for JEV. Supplementary Table S2 summarizes the number of up- and downregulated proteins reported in each experiment.

### Datasets for interactomic analyses

Infectious diseases are the result of molecular crosstalk between hosts and their pathogens. Literature searches with keywords “protein–protein interaction” and “human and virus” were performed to collect interactomic data. Data for interactomic analysis were manually extracted by reading the literature selected from these literature searches. This crosstalk mediated by host–pathogen protein interactions was collected for the five viruses (Supplementary Table S3).

### Multiple omics analyses

We determined the signature molecules by multiple omics analyses, selecting the intersection of signature gene products by transcriptomic analysis, signature proteins by proteomic analysis and human–viral PPIs by interactomic analysis. The GSEA method was used to find statistically significant pathways from the Molecular Signatures Database (MsigDB)^17^ and Pathway Ontology (PWO)^18^ for differentially expressed genes, as presented in a previous study^10^. A hypergeometric test was applied to significant gene groups between infected and normal samples to identify the infection-specific pathways in the MsigDB and PWO with *p* < 0.05. We performed GSEA for the upregulated (overexpressed) molecules with the molecular signatures of the canonical pathway (c2.cp.v6.2) and PWO (http://purl.bioontology.org/ontology/PW). Upregulated signature pathways were selected by combined transcriptomic and proteomic analyses.

### Drug repositioning candidates

The CMap method^19^ was used with the transcriptomic analysis to identify any inverse drug-disease relationships. For individual gene expression datasets, the probe list transformed from upregulated and downregulated signature genes between infected and normal samples was applied to CMap to identify any inverse drug-disease relationships^10^. CMap compares the gene expression profile of cells before and after exposure to compounds and quantitatively assesses changes in transcriptome profiles caused by active compounds. Comparisons of transcriptomic profiles between drug response and disease phenotypes can reveal underlying pathological processes. In our computational method, the differential gene expression profiles between disease and control samples (diseased versus healthy subjects) were used as the main single data layer to select drug candidates and is a summary of the effects of the drugs (i.e., genes upregulated in the disease profile were downregulated in the drug profile and vice versa). The effect of a drug on gene transcription levels was assumed to be opposite to the effect of the disease. The threshold of significance for each drug was set at *p* < 0.1 using the permutated results. For multiple datasets, i.e., DENV and WNV, the union of each drug candidate from individual gene expression datasets was selected as the drug candidates in the viral infection.

STITCH 5.0^20^ was used to find interactions between chemical compounds and proteins as an interaction network. We set the acceptable “combined score” to > 0.7 to ensure a high level of confidence for the interaction^10^. The obtained interaction network accepted only potential drug candidates and extracted the interactions between chemical compounds and significant proteins and the human proteins involved in human–viral PPIs. Drug candidates selected were those that interacted directly with common signature proteins, which was based on omics analyses, and were extracted with the proteins participating in signature pathways.

### Network analysis

We have performed a network analysis to explore the relationship between the drug candidates and protein interactions for the five infectious diseases. Human proteins identified to interact with viral proteins experimentally were extracted. We explored drug candidates that inhibit two classes of PPIs: host–pathogen and host–host interactions to investigate the connection among the five infections in a human protein–protein network. In considering host–host interactions, we selected human proteins connected directly to those disease-related human proteins from the human protein–protein interaction network, Human Protein Reference Database (HPRD release 9)^13^. HPRD contains binary data for 38,775 interactions and 9,561 human proteins. The identified proteins associated with the five viruses were projected onto the HPRD to characterize the network properties of the relationship between proteins and diseases, and to explore how combined treatment of these diseases could be achieved by drug repositioning. This was accomplished by introducing “extended signature proteins”, which considers proteins directly connected to “common signature proteins” (union of proteome and interactome). We counted the connectivity of proteins (degree), which is the number of binding partner proteins in the HPRD to investigate characteristic hub proteins. In addition, we identified “shared proteins”, which are overlapping proteins of the common and extended signature proteins in the five infections. Computing the shortest distances among the shared target proteins in the HPRD created a minimum network. The network then added bridge proteins that linked shared proteins to the five diseases.

## Results and discussion

### Signature molecules by multiple omics analyses

We compared signature genes by transcriptomic analysis, signature proteins by proteomic analysis and human-viral PPIs. Transcriptomic analysis identified 1000 signature genes (500 upregulated and 500 downregulated genes based on FC values) between infected and uninfected states for the five viruses. Upregulated genes were used for omics analysis. For proteomics data, up- and downregulated proteins were extracted from our literature searches. Upregulated molecules in transcriptomic and proteomic analyses were used for further omics analysis. For interactomic data, we used two classes of PPIs: human (disease related proteins)–viral and human (disease related proteins)–human interactions, as described in the Methods section. The numbers of human proteins in human–viral interactions for DENV, ZIKV, WNV, CHIV and JEV were 345, 518, 34, 159 and 26, respectively. The human-infectious diseases PPIs include the two classes of PPIs for omics analysis.

**Figure 2:**
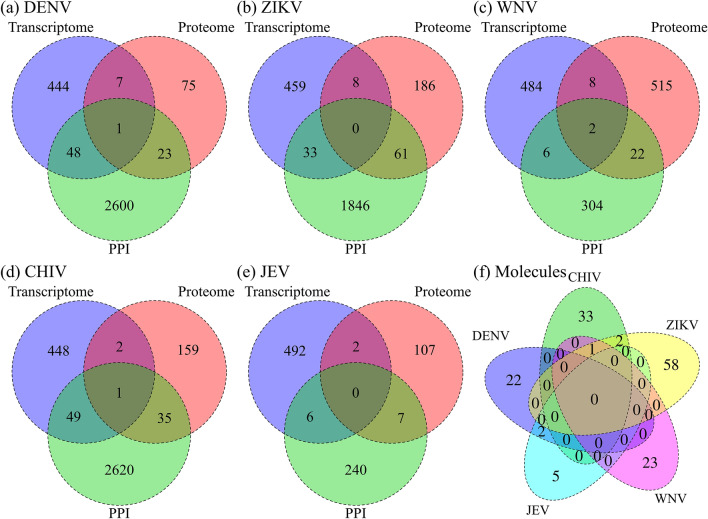
Signature genes and proteins by multiple omics analyses. Venn diagrams show the overlap of signature genes and proteins by multiple omics analyses for the five viruses: (**a**) DENV, (**b**) ZIKV, (**c**) WNV, (**d**) CHIV and (**e**) JEV. The number of signature genes obtained from transcriptomic data with significant differences is shown in the blue circle for infections. The number of signature proteins obtained from proteomics data is shown in the orange circle. The number of human proteins that interact with viral proteins in human–virus PPIs is shown in the green circle. (**f**) Venn diagram of common signature proteins, which represent the intersection between proteome and PPIs for the five viral infections. The total number of the unique proteins was 146 for the five viral infections.

For DENV, the 500 signature genes, 106 signature proteins identified by the proteomic analysis and 2672 human proteins identified as human–DENV PPIs are compared in Fig. 2a. Forty-nine proteins overlapped between the signature gene products and human proteins from the human–DENV PPIs, and 24 proteins overlapped between the signature proteins and the human proteins from the human–DENV PPI network. The intersection of the three sets among transcriptomic, proteomic and interactomic analyses contains a protein (HSP90B1). Protein abbreviations are listed in Supplementary Information. Venn diagrams comparing signature genes, signature proteins and human proteins in human–viral PPI networks for ZIKV, WNV, CHIV and JEV infections are shown in Fig. 2b–e. At the intersection, two proteins (PML, STAT1) for WNV and one protein (SDK2) for CHIV were found.

We focused on the “common signature proteins” at the intersections of proteome and interactome analyses. The number of common signature proteins in DENV, ZIKV, WNV, CHIV and JEV was 24, 61, 24, 36 and 7, respectively. The total number of unique proteins was 146. Figure 2f shows the relationship among the five viruses. Only one protein, NPM1 (nucleophosmin), which is a multifunctional chaperone, was shared by three viruses among neurological viruses (ZIKV, WNV) and the inflammatory arthritis virus (CHIV). Nucleophosmin activates chromatin transcription in an acetylation-dependent manner and is important for restricting virus replication in host–virus interactions, especially in ZIKV^21^, CHIV and WNV infections^22,23^. One protein (RTN4) was common between DENV and ZIKV and three proteins (NCL, RAN and VCL) were common between ZIKV and CHIV. Most of the signature molecules were unique to infections. Supplementary Fig. S1 shows a comparison of the signature molecules for the five viruses derived from transcriptomic, proteomic and interactomic analyses. In Fig. 2f, no common signature proteins from neurological complications (ZIKV, WNV, JEV) were observed at the intersections for the three viral diseases. Thus, it may be difficult to identify common signature molecules linked to phenotypes by simply selecting the intersection of the three layers of omics analyses.

### Signature pathways by multiple omics analyses

**Figure 3:**
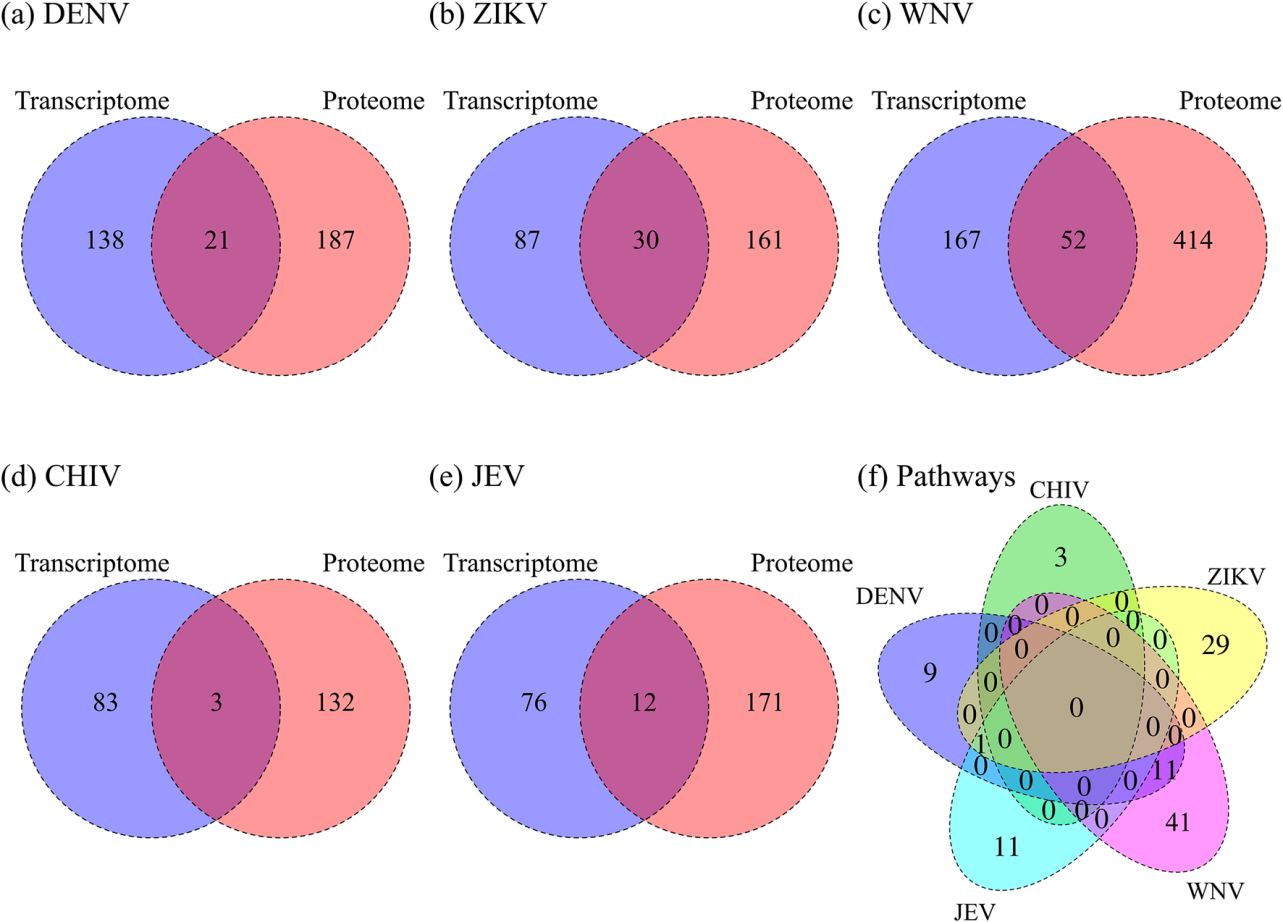
Signature pathways by multiple omics analyses. Venn diagrams show the overlap of the signature pathways by GSEA analysis for (**a**) DENV, (**b**) ZIKV, (**c**) WNV, (**d**) CHIV and (**e**) JEV. The number of significant pathways of the transcriptome and proteome are shown in the blue and orange circles, respectively. (**f**) Venn diagram of the signature pathways, which are intersections derived from the transcriptomic and proteomic analyses for the five viral infections.

We analyzed the five viral infections in coarse-grained pathway units by using GSEA with upregulated molecules for the transcriptomic and proteomic data. Venn diagrams of pathways for the five viral infections are shown in Fig. 3a–e. In addition to the molecular level, in which the gene profile shows the opposite pattern when selecting drug candidates in CMap, we conducted a network analysis to determine the activity status of signal transduction pathways and transcription factor networks in the diseases. The results of the pathway analysis were used in our filtering method described below.

Using the Venn diagram in Fig. 3f, the pathways that were unique to each viral infection and shared among the five viral infections were identified. The approach revealed that it is difficult to find a common upgraded pathway for those viruses by coarse-grained pathway analysis. Only the "disease pathway" in PWO was common to ZIKV, JEV and DENV for hemorrhagic syndrome. The results of this analysis are used for filtering, as described below. Supplementary Fig. S2 shows a comparison of the signature pathways for the five viral infections for transcriptomic, proteomic and interactomic analyses. The total number of unique pathways identified was 105.

### Drug candidates by multiple omics analyses

**Figure 4:**
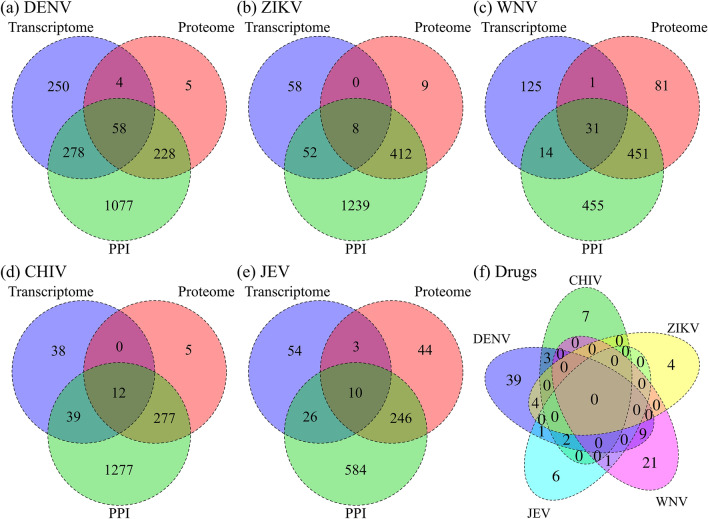
Drug candidates by multiple omics analyses. The numbers of drug candidates identified by the transcriptomic, proteomic and interactomic analyses are shown in blue, orange and green circles, respectively. The numbers of drug candidates that overlap between each analysis type are also shown: (**a**) DENV infection, (**b**) ZIKV infection, (**c**) WNV infection, (**d**) CHIV infection and (**e**) JEV infection. (**f**) The drug candidates of the intersection of the three omics analyses in five viral infections are shown. The numbers of drugs at the intersection of the three layers for DENV, ZIKV, WNV, CHIV and JEV are 58, 8, 31, 12 and 10, respectively. The total number of unique drugs was 97.

We identified drug candidates for each infection based on omics analyses. From transcriptomic analysis, the 300 most upregulated and the 300 most downregulated signature genes for the five diseases obtained from GEO data sets that were compatible with the HG-U133A platform of CMap were used to query the CMap system. The rank matrix of CMap was built by fold change based on differential expression profiles. The up- and downregulated genes selected by fold change were used as differentially expressed signature genes for drug repositioning. STITCH 5.0 was used to search for drug candidates that interact with the signature proteins from proteomes and human–viral PPIs. Figure 4a–e shows drug candidates from multiple omics analyses. For DENV, 588 compounds with statistical significance (*p* < 0.1) using the CMap permutated results were identified as the union of each dataset (GSE50698: 125; GSE23986: 100; GSE51808: 119; GSE34628: 22; GSE9378: 118; GSE18090: 128; GSE40628: 161; GSE38246: 160; GSE25226: 78; GSE58278: 67). By searching STITCH for drug candidates based on the proteomic data, we found 293 drug candidates that interacted with 106 significant proteins. For drug candidates from the interactomic data, we found 1,641 drug candidates in STITCH that interacted with 2672 human proteins identified in the human–viral PPIs. Finally, 58 drug candidates were identified that overlapped over the analyses of the three layers. For ZIKV, 118 compounds were identified using the CMap as GSE98889. Four-hundred and twenty-nine drug candidates interacted with 255 significant proteins, and STICH identified 1711 drug candidates that interacted with 1940 human proteins in PPIs. Eight drug candidates were shared over the analyses of the three layers. CMap detected 171 compounds as the union of each dataset (GSE46681: 104; GSE30719: 117) for WNV. Here, 564 drug candidates interacted with 547 significant proteins and STITCH identified 951 drug candidates that interacted with 334 human proteins in PPIs. Thirty-one drug candidates were shared over the analyses of the three layers. For CHIV, 89 compounds were identified as GSE49985. Two-hundred and ninety-four drug candidates interacted with 197 significant proteins and 1605 drug candidates were identified by STITCH to interact with 2705 human proteins in PPIs. Finally, we identified 12 drug candidates that were shared over the analyses of the three layers. For JEV, 93 compounds were identified as GSE57330. Three-hundred and three drug candidates interacted with 116 significant proteins and 866 drug candidates interacted with 253 human proteins in PPIs. Ten drug candidates were detected over the analyses of the three layers.

Drugs common in up to three infectious diseases were found by selecting drug candidates for each infectious disease. Figure 4f shows the drug candidates at the intersection of the three omics analyses for the five diseases. Supplementary Fig. S3 compares drug candidates for the five viral infections from transcriptomic, proteomic and interactomic analyses. Supplementary Fig. S4 shows the multi-omics analyses for three diseases (ZIKV, WNV and JEV) that cause neurological complications. Only one drug was found at the intersection of WNV and JEV. These results show that it may be difficult to identify common signature molecules and drugs linked to phenotypes.

### Filtered drug candidates for the five viral infections

Drug candidates for the five infections were selected by our filtering method^10^. In brief, we have filtered and selected drug candidates based on common proteins (union of proteins in Fig. 2), common pathways (union of pathways in Fig. 3) and common drugs (union of drugs in Fig. 4) of the three layers. For DENV (Fig. 4a), 56 drugs were identified as the intersection of the drug candidates by omics analyses. This process yielded 24 common proteins (union of proteome and PPI in Fig. 2a), 21 common pathways (union of transcriptome and proteome in Fig. 3a) and 58 drug candidates. Supplementary Fig. S5 shows the filtering method process for identifying drugs against DENV. We identified eight potent drugs for dengue hemorrhagic fever (DHF) previously^10^. Seven of the eight drugs against DHF are also found in the 56 DENV drug candidates. Using the same method described above, four drug candidates against ZIKV infection, 25 drug candidates against WNV infection, six drug candidates against CHIV infection and eight drug candidates against JEV infection were obtained (Supplementary Figs. S6–S9). Supplementary Table S4 lists all drug candidates that were identified by omics analyses for the five viruses. Supplementary Fig. S10 shows the flow diagram for the union of all results obtained for the five viral infections. A total of 77 unique drug candidates were identified. Supplementary Fig. S11a shows a comparison of the 77 drug candidates for the five viral infections. For the neurological diseases, we found 36 unique drug candidates and their virus targets are shown in Supplementary Fig. S11b.

We evaluated the computationally identified common drugs with previously reported drugs. A total of 20 drug candidates were identified to be common to two or three infections. Trichostatin A and vorinostat were identified to be common to DENV, CHIV and JEV infections; acetylsalicylic acid, dinoprostone, glibenclamide and trifluoperazine were common to DENV and ZIKV; dinoprost, genistein, LY-294002, melatonin, ouabain, rosiglitazone, SB-203580, sirolimus and troglitazone were common to DENV and WNV; alvespimycin, orlistat and tanespimycin were common to DENV and CHIV; ascorbic acid was common to DENV and JEV; and staurosporine was common to WNV and JEV. Seven of the 20 drug candidates that appear to be common for two viruses have been reported in experimental drug repositioning studies for one of the viruses. Trichostatin A and vorinostat are histone deacetylase (HDAC) inhibitors. Using high concentrations of HDAC inhibitors caused a significant reduction in cytokine production in DENV2 infections^24^. Among HDAC inhibitors, selective HDAC6 inhibitors concentration-dependently inhibited JEV-induced cytopathic effects and apoptosis, as well as reduce virus yield in human cerebellar medulloblastoma cells^25^. Genistein treatment significantly reduced uptake of h3H5-opsonized DENV in a concentration-dependent manner^26^. LY-294002 inhibits PI3K activation, and greatly enhanced virus-induced cytopathic effects, even at an early stage of infection^27^. Ouabain is a reducing agent that cleaves disulfide linkages between two polypeptide chains that are essential for T cell helper cytokine activity^28^. SB-203580 is a p38 MAPK inhibitor that has been shown to reduce DENV-induced liver injury in a mouse model^29^. Sirolimus, an mTOR pathway inhibitor and a potential inhibitor of CypA, significantly reduced yellow fever virus replication in immunofluorescence and viral plaque assays^30^. Orlistat is a potential broad-spectrum agent against multiple mosquito transmitted viruses^31^. Valproic acid was found to reduce significantly the production of all cytokines produced in DENV2 infections^24^. A patient with CHIV fever was treated with high doses of intravenous vitamin C over two days, and symptoms resolved after infusions without any side effects^32^. The above results indicate that our multi-omics analyses rationally identified potential drug candidates for treating multiple viral infections.

### Network analyses by the projection of the human protein–protein interaction network

**Figure 5:**
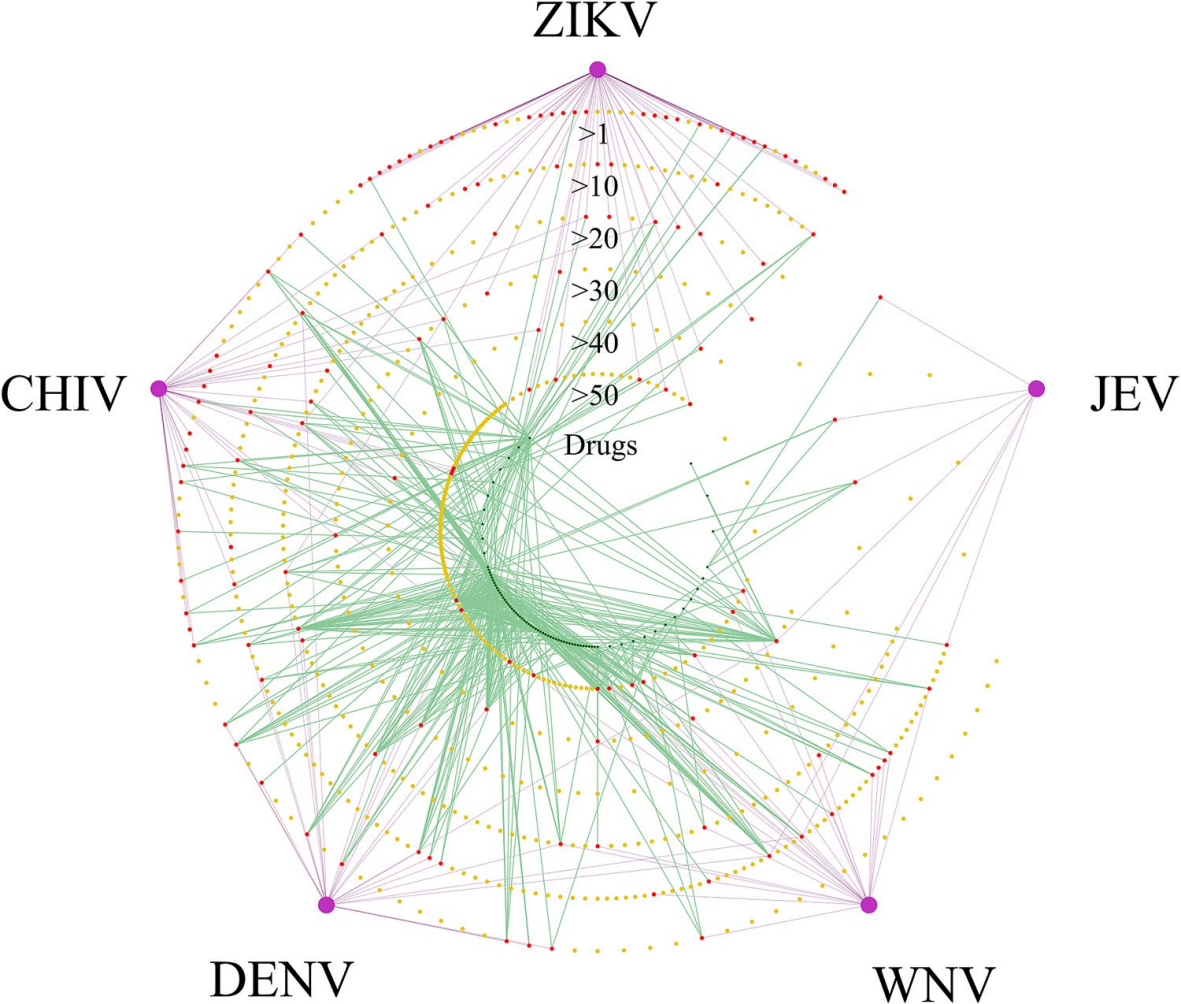
The network shows the relationship among drug candidates, signature proteins and the five viruses. The concentric circles show the network of diseases (purple circles), proteins (red and yellow circles) and drugs (green circles). The numbers of extended signature proteins (yellow) for DENV, ZIKV, WNV, CHIV and JEV are 262, 351, 352, 289 and 99, respectively. The degrees of the proteins calculated from the PPI network in the HPRD are divided into 0 to 10, 10 to 20, 20 to 30, 30 to 40, 40 to 50 and 50 or greater. The circularly distributed proteins with smaller radii indicate higher degrees. The numbers of common (red) and extended (yellow) proteins counting from the outer circle inwards are 32, 34, 40, 46, 48 and 57 for common proteins and 47, 54, 71, 94, 116 and 190 for extended proteins. The purple edge shows the connection between the disease and common proteins. The green edge shows the signature protein-drug candidate interactions.

In the previous sections, we identified 77 drug candidates (Supplementary Table S4) and 146 common signature proteins (Fig. 2f) for the five infections using multiple omics analyses. The results showed that identifying common drugs for the five viral infections was not possible (Fig. 4f, Supplementary Figs. S4c and S11), regardless of the clinical outcomes or that these compounds target the same viral family. We then performed a network analysis to investigate the drug candidates against the five infectious diseases by exploring the relevance between those identified drugs and diseases through protein interactions. The network analysis was used to find how the candidate drugs listed are related to the human protein network in the diseases. The signature proteins from the five viruses were projected onto the human protein network of the HPRD. Figure 5 shows the drug candidates and the common signature proteins presented in a network diagram for the five viruses. The degree of common and extended signature proteins was calculated as the number of connections to proteins based on the human PPI network from the HPRD. Beside WNV, the average degree of common proteins for each infection was lower than the average degree of extended proteins. The degree of common proteins was found to concentrate at intermediate degree values (i.e., 10 < degree ≤ 150). Supplementary Fig. S12 shows the frequency distribution of the degree on the HPRD for common signature proteins from each virus. The median degree of the signature proteins for each virus is moderately higher than that of the overall HPRD degree distribution. The overall HPRD has a larger tail, whereas the degree distribution of each virus does not have a tail. This indicates that signature proteins that are closely related to infectious diseases do not show characteristics of a high-degree hub protein but a moderate-degree hub protein. We also investigated the networks of the extended proteins that are involved in human host–host PPIs for each infectious disease. Extended proteins are not connected directly to viral proteins (disease related proteins) but to common proteins that are selected from signature proteins in proteomic analysis and disease related proteins in interactomic analysis. The relationship of the drug candidates and the extended proteins in a network diagram for the five viruses is shown in Supplementary Fig. S13. The number of connectivities among drugs and extended proteins is much greater than among drugs and common proteins for the five diseases.

**Figure 6:**
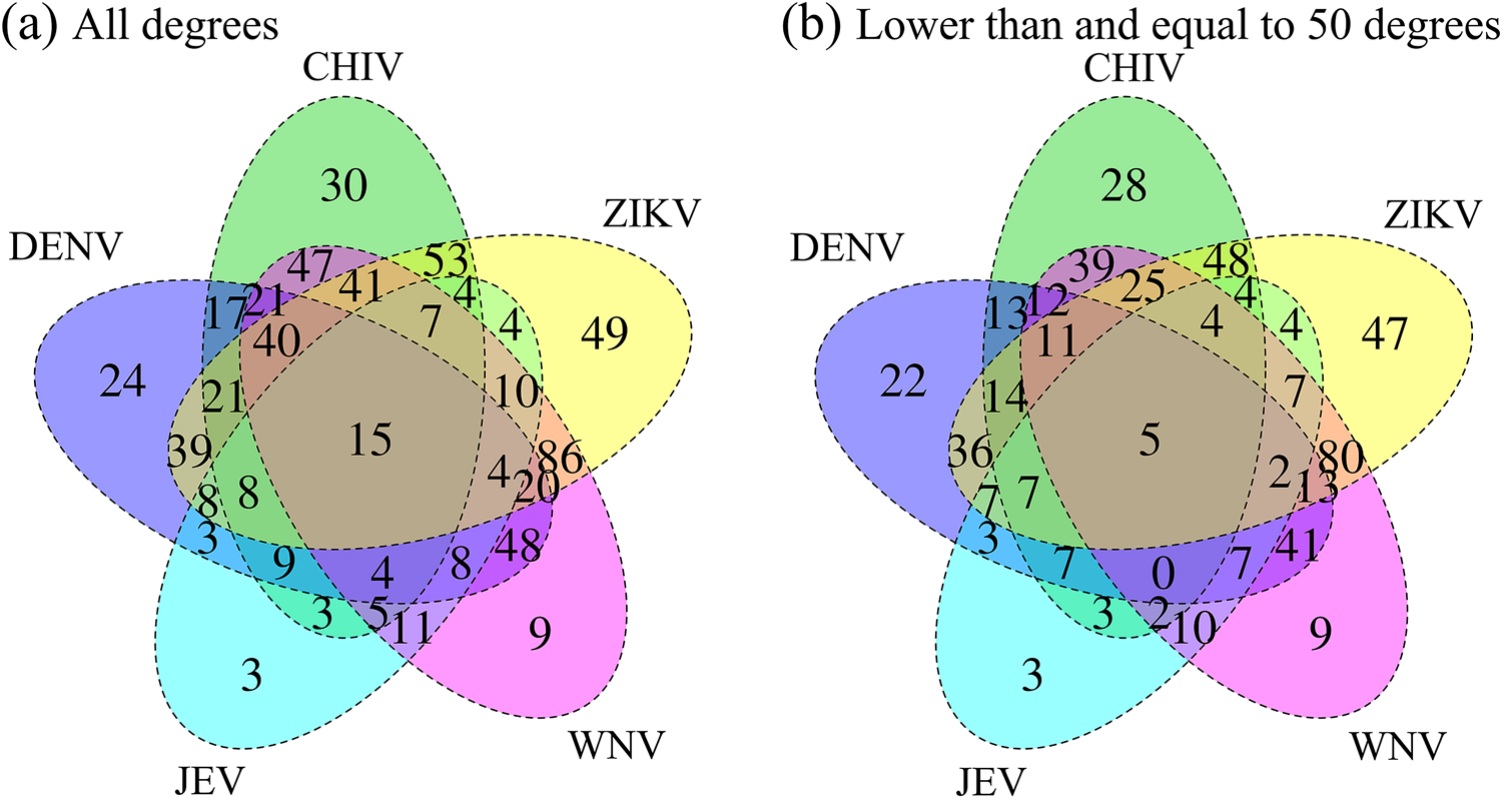
Venn diagrams show the shared numbers of the common and extended signature proteins for the five viral infections. Common and extended signature protein numbers with all degrees are shown in (**a**) and those proteins with lower than and equal to 50 degrees are given in (**b**).

**Figure 7:**
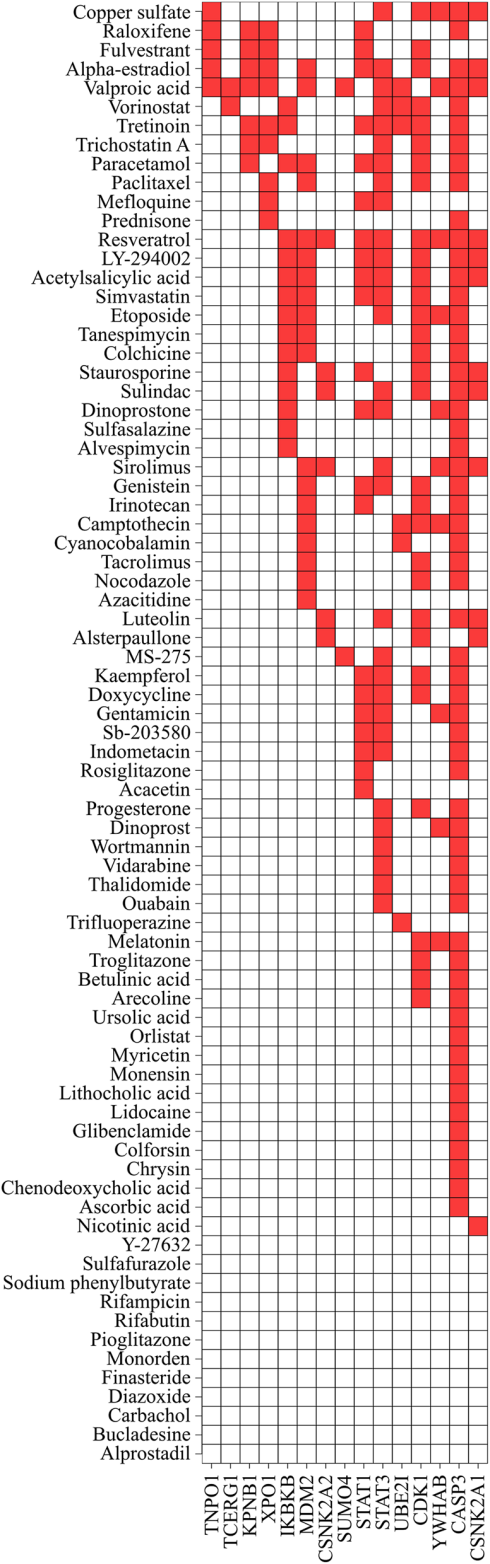
Two-dimensional matrix for drug-protein interactions between 77 filtered drug candidates and 15 shared proteins. Rows and columns represent 77 filtered drug candidates and 15 shared proteins, respectively. The proteins in the figure are arranged from the left in ascending order of the degree in the HPRD. Red squares indicate interactions between proteins and drug candidates found by STITCH.

Figure 6 shows Venn diagrams of common and extended signature proteins for the five virus infections. Considering all degrees of the proteins, 15 shared proteins overlap among the five infections (Fig. 6a). For degrees with lower than and equal to 50 proteins (five outer circles in Fig. 5), which indicate less hub-protein properties, five shared target proteins, TNPO1, TCERG1, KPNB1, XPO1 and IKBKB, were identified (Fig. 6b). Transportin-1 (TNPO1) functions in nuclear protein import as a nuclear transport receptor and serves as a receptor for nuclear localization signals in cargo substrates. Exportin-1 (XPO1) mediates the nuclear export of cellular proteins bearing a leucine-rich nuclear export signal and RNAs. Viruses can use the classical importin-α/β pathway, importin-β directly, the nuclear pore complex, or transport through a PY-nuclear localization signal for nuclear import as well as exportin-1 for nuclear export^33^. Transcription elongation regulator 1 (TCERG1) binds RNA polymerase II and inhibits the elongation of transcripts from target promoters. Importin subunit beta-1 (KPNB1) functions in nuclear protein import, either in association with an adapter protein, like an importin-alpha subunit, which binds to nuclear localization signals in cargo substrates, or by acting as an autonomous nuclear transport receptor. Inhibitor of nuclear factor kappa-B kinase subunit beta (IKBKB) is activated by multiple stimuli such as inflammatory cytokines, bacterial or viral products, DNA damage or other cellular stresses. For neuropathies, 36 shared proteins overlap among three infections after taking into consideration all degrees of the proteins (Supplementary Fig. S14a). For degrees with lower than and equal to 50 proteins, 18 shared proteins among three diseases were found (Supplementary Fig. S14b). Table 1 summarizes the degree of distribution in the HPRD of the 15 shared proteins. Figure 7 shows a two-dimensional matrix of the interactions between the 15 shared proteins in the five diseases and the 77 filtered drug candidates. The number of drug candidates that interact with a protein increased as the order of a protein increased. Sixty of the 77 filtered drug candidates targeted two shared proteins with 130 or more degrees. There is a large number of drug candidates that target high-degree proteins. The properties of high-degree proteins facilitate interactions with a wide variety of drugs, indicating that interactions with the drug may have low specificity and many side effects.

**Table 1:** Filtered drug candidates with shared target protein degrees for the five viral infections.

Size of degree	Number of shared targets	Name of shared target proteins	The number of drug candidates
0 < deg. ≤ 10	0		0
10 < deg. ≤ 20	1	TNPO1	5
20 < deg. < = 30	1	TCERG1	2
30 < deg. ≤ 40	0		0
40 < deg. ≤ 50	3	IKBKB,KPNB1,XPO1	23
50 < deg. ≤ 60	1	MDM2	19
60 < deg. ≤ 70	0		0
70 < deg. ≤ 80	3	CSNK2A2,STAT1,SUMO4	27
80 < deg. ≤ 90	0		0
90 < deg. ≤ 100	0		0
100 < deg. ≤ 110	2	STAT3,UBE2I	34
110 < deg. ≤ 120	0		0
120 < deg. ≤ 130	2	CDK1,YWHAB	36
130 < deg. ≤ 140	1	CASP3	58
140 < deg. ≤ 150	0		0
150 < deg	1	CSNK2A1	12

**Figure 8:**
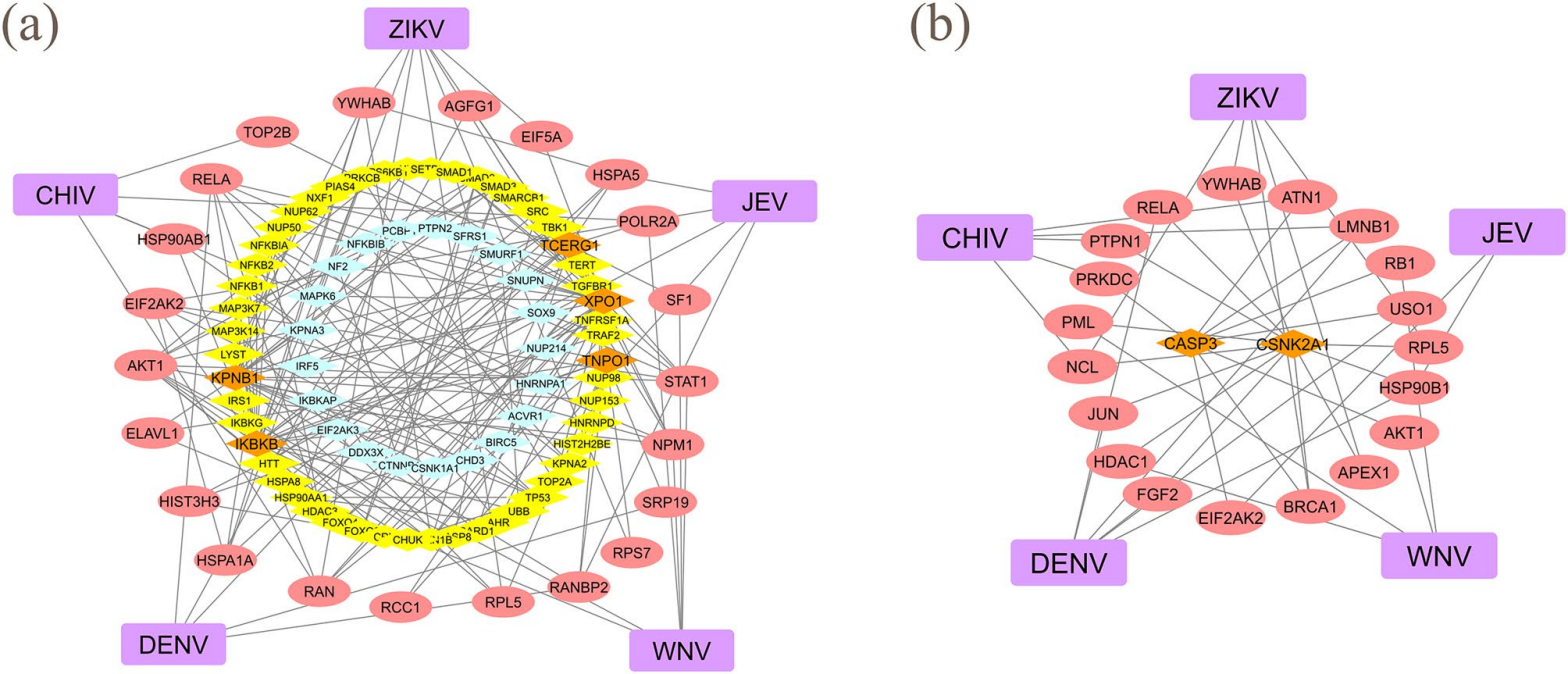
(**a**) Minimum network with a low degree of the target proteins. The target proteins, which are shared proteins TNPO1, TCERG1, IKBKB, KPNB1 and XPO1 with 50 or less degrees, are shown as orange diamonds. Common (red ellipses) and extended (yellow diamonds) signature proteins in this network are connected to the five viral infections (purple round squares). Twenty-one added proteins are shown as light blue diamonds. (**b**) Minimum network with a high degree of the target proteins. The target proteins, which are shared proteins CASP3 and SNK2A1 with greater than 130 degrees, are shown as orange diamonds.

We investigated the connectivity of the shared proteins dependence on the degree of 15 proteins (Fig. 6a). By computing the shortest distances among the shared target proteins in the HPRD, a minimum network was created by adding bridge proteins from the HPRD where necessary. The minimum network ensured that shared proteins are linked to the five diseases; thus, connecting these proteins created edges among the common and extended proteins in the five diseases. For example, focusing on the five shared proteins (low hub proteins) with 50 or less degrees in Fig. 6b, we found that the addition of 21 bridge proteins were required to create the network among the five proteins. Figure 8a shows the network of common, extended and 21 added proteins. For the largest degree of proteins in Table 1, the shared target proteins in the five infections with 130 or more degrees are two proteins (high hub proteins), CASP3 and CSNK2A1, which are known hub proteins in PPIs and interact with 60 drug candidates. Interestingly, the network consists of two extended proteins and common proteins that are connected directly to viral proteins (human–viral interactions) in each infection. By comparing the low degree of shared proteins in Fig. 8a, the two hub proteins clearly showed crosstalk mediated by host–pathogen proteins. The drugs target extended proteins that are proximal to the diseases. Comparison of minimum networks between Fig. 8a and b showed that the hub proteins in Fig. 8b are located in a small neighborhood for the five diseases. This observation suggests that drugs targeting the shared proteins linked to the five diseases may potentially treat multiple infections. Fifty-one of the 60 drug candidates were non-anticancer agents.

We further investigated the 51 drug candidates by examining their interaction with 50 or less degree proteins by considering the coverage of low hub (TNPO1, TCERG1, KPNB1, XPO1 and IKBKB) and hub proteins in the network analysis. We have classified 17 of the 51 drug candidates (acetylsalicylic acid, alpha-estradiol, alvespimycin, colchicine, copper sulfate, dinoprostone, LY-294002, paracetamol, resveratrol, simvastatin, staurosporine, sulfasalazine, sulindac, tanespimycin, tretinoin, trichostatin A, valproic acid). One of 17 drug candidates (trichostatin A) was determined to be a common drug candidate for three infections (DENV, CHIV and JEV) by the filtering method using multi-omics analyses. Trichostatin A is a histone deacetylase inhibitor and its activity against DENV and JEV infections has been examined^24,25^. Six of the 17 drug candidates (acetylsalicylic acid, alvespimycin, dinoprostone, LY-294002, staurosporine, tanespimycin) were determined to be common drug candidates for any two infections. Ten drug candidates (alpha-estradiol, colchicine, copper sulfate, paracetamol, resveratrol, simvastatin, sulfasalazine, sulindac, tretinoin, valproic acid) were found to be repositioning drugs for one infection. Three of the ten drugs (paracetamol, resveratrol, valproic acid) are potential agents against DENV infection. Supplementary Table S4 summarizes the drug candidates identified by multiple omics and network analyses of the 15 proteins.

We have identified 77 drug candidates by our filtering method that may be effective against one to three viral infections. We constructed a minimum network of PPIs that connected the five diseases. Taking into consideration the degree of the proteins, we computationally selected 17 drugs that targeted low hub and hub proteins on the network (Fig. 7). Investigating the relationship between drugs and diseases via PPI indicated that 17 drugs may inhibit all diseases through low and high hub proteins. The computationally identified drugs were validated by experimental evidence from literature where these drugs have been tested and clinical trial information. This method combining multiple omics data with network analysis may be useful in finding compounds against diseases where experimental reports are relatively sparse, such as JEV. Limitations associated with this study can arise when the number of available data is low and datasets show large variability. Accumulation of high-quality data should improve this drug selection method. In addition to the omics data provided here, integration with other omics data such as genome-wide association studies (GWAS), metabolomics, genomics, phosphoromics and methylomics can be considered in future studies.

## Conclusion

By combining multiple omics analyses based on experimentally obtained molecular profiling and network analysis of human PPIs, we have identified potential drug repositioning candidates for treating mosquito-borne DENV, ZIKV, WNV, CHIV and JEV infections. The signature molecules, pathways and the viral–human PPIs were determined based on large-scale intergradation of experimentally measured data for five viral infections. By applying our filtering methods with signature molecules and pathways, drug candidates to treat each viral infection were identified. A total of 77 drug candidates and 146 proteins were reported for the five infections using multiple omics analyses.

Network analysis of signature proteins was also performed to characterize the drugs for the five infectious diseases using PPI networks. A network-represented relationship among drug candidates, infections and proteins was created to study the connectivity of proteins in the HPRD. By projecting proteins onto the HPRD, the degree of disease-related proteins was found to be relatively low. This indicates that disease-related proteins with a close relationship to viral infections do not present characteristics of a high-degree hub protein but a moderate-degree hub protein. Fifteen proteins that were connected to the five infections were targeted by 77 drug candidates. By analyzing the degree of those proteins, we have classified 51 drug repositioning candidates that interact with hub proteins for the five diseases and 17 out of those 51 candidates also interact with low hub proteins. We compared the computationally identified drugs with previously reported drugs and clinical trial data. Four of these drugs, trichostatin A, valproic acid, resveratrol and paracetamol, have been reported as effective treatments for flavivirus-induced diseases. This observation illustrates that our computational approach using omics data such as public gene expression microarray data, proteomics data and PPIs can identify potential drugs.

The presented computational method should be an effective approach to identify novel drug candidates that target multiple, related diseases. The large-scale intergradation of experimentally measured data and network analysis makes it possible to draw a comprehensive view of biological processes in multiple diseases. This study should aid researchers tackling emerging viral disease outbreaks that are transmitted by mosquitoes.

## Supplementary Information

Supplementary Information.

Supplementary Table S4.
