# Supplementary material for: Application of multiple omics and network projection analyses to drug repositioning for pathogenic mosquito-borne viruses
Source: Sci Rep. 2021 May 12;11:10136. doi: 10.1038/s41598-021-89171-x (PMC8115341; doi:10.1038/s41598-021-89171-x)
Supplement: Supplementary file 1 — Supplementary Information. [file 41598_2021_89171_MOESM1_ESM.pdf]

Supplementary Information

Application of multiple omics and network projection analyses to drug repositioning for pathogenic mosquito-borne viruses

Takayuki Amemiya[1,2], Katsuhisa Horimoto[2] & Kazuhiko Fukui[1,2*]

[1]*Cellular and Molecular Biotechnology Research Institute, National Institute of Advanced Industrial Science and Technology (AIST), Tokyo 135-0064, Japan*

[2]*Molecular Profiling Research Center for Drug Discovery (molprof), National Institute of Advanced Industrial Science and Technology (AIST), Tokyo 135-0064, Japan*

E-mail: k-fukui@aist.go.jp

Abbreviations

HSP90B1	Endoplasmin
PML	Protein PML
STAT1	Signal transducer and activator of transcription 1-alpha/beta
SDK2	Protein sidekick-2
NPM1	Nucleophosmin
RTN4	Reticulon-4
NCL	Nucleolin
RAN	GTP-binding nuclear protein Ran
VCL	Vinculin
TNPO1	Transportin-1
TCERG1	Transcription elongation regulator 1
KPNB1	Importin subunit beta-1
XPO1	Exportin-1
IKBKB	Inhibitor of nuclear factor kappa-B kinase subunit beta
CASP3	Caspase-3
CSNK2A1	Casein kinase II subunit alpha

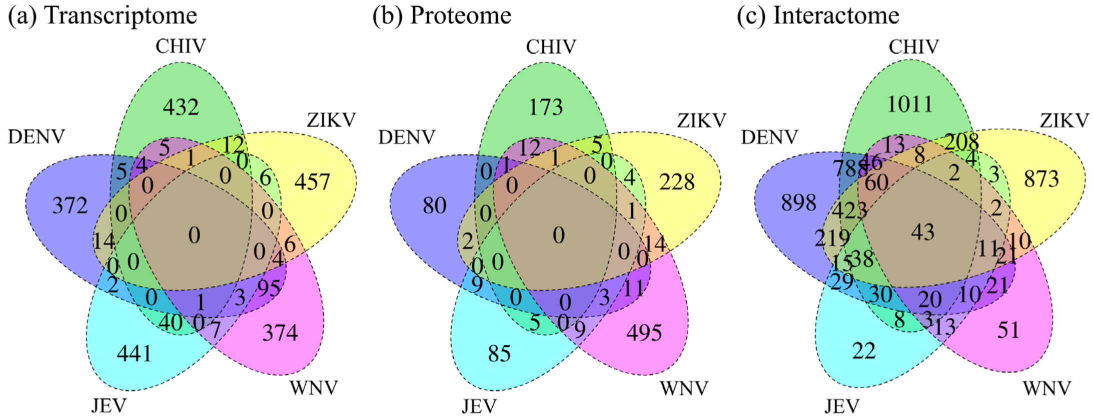

Supplementary Fig. S1. Venn diagrams show the shared numbers of signature genes and proteins for the five viral infections. Signature genes and proteins taken from (a) transcriptomic, (b) proteomic and (c) interactomic analyses for each infection.

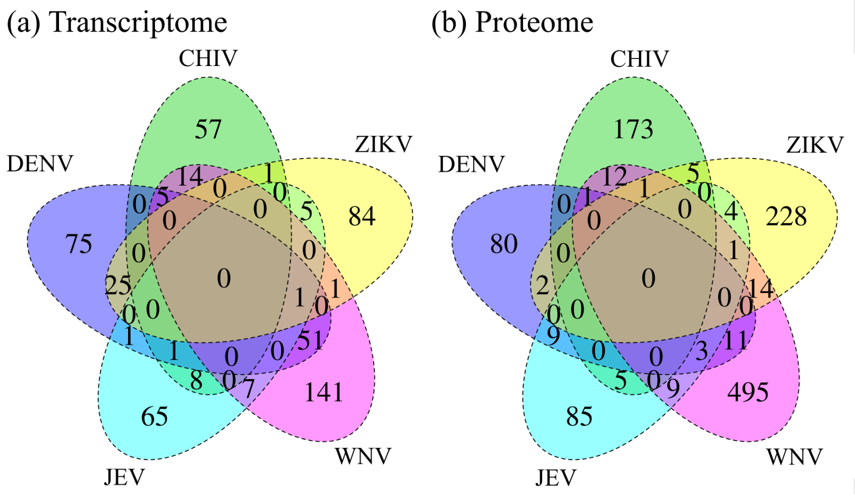

Supplementary Fig. S2. Venn diagrams show the shared numbers of signature pathways for the five viral infections. Signature pathways taken from (a) transcriptomic and (b) proteomic analyses for each infection.

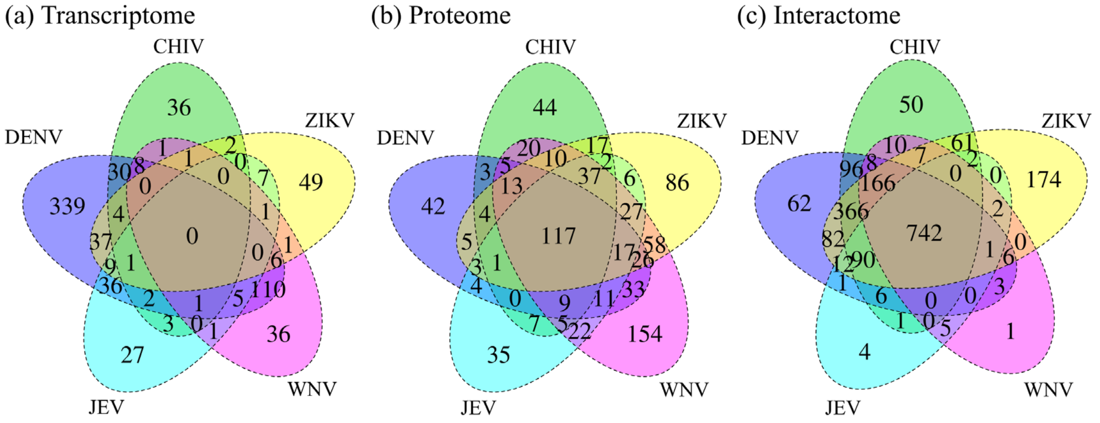

Supplementary Fig. S3. Venn diagrams show the shared numbers of drug candidates for the five viral infections. Drug candidates taken from (a) transcriptomic, (b) proteomic and (c) interactomic analyses for each infection.

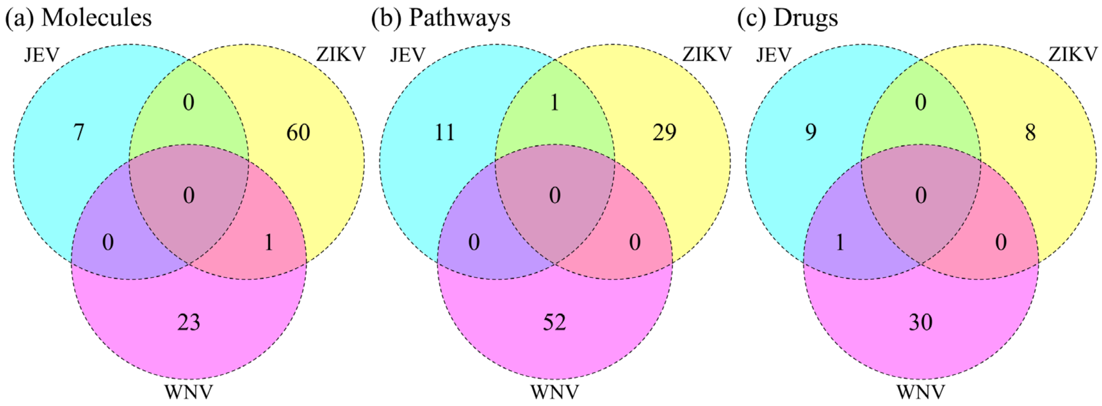

Supplementary Fig. S4. Results by multiple omics analyses for the neurological complications. The Venn diagrams show the shared numbers of (**a**) signature genes and proteins, (**b**) signature pathways and (**c**) drug candidates.

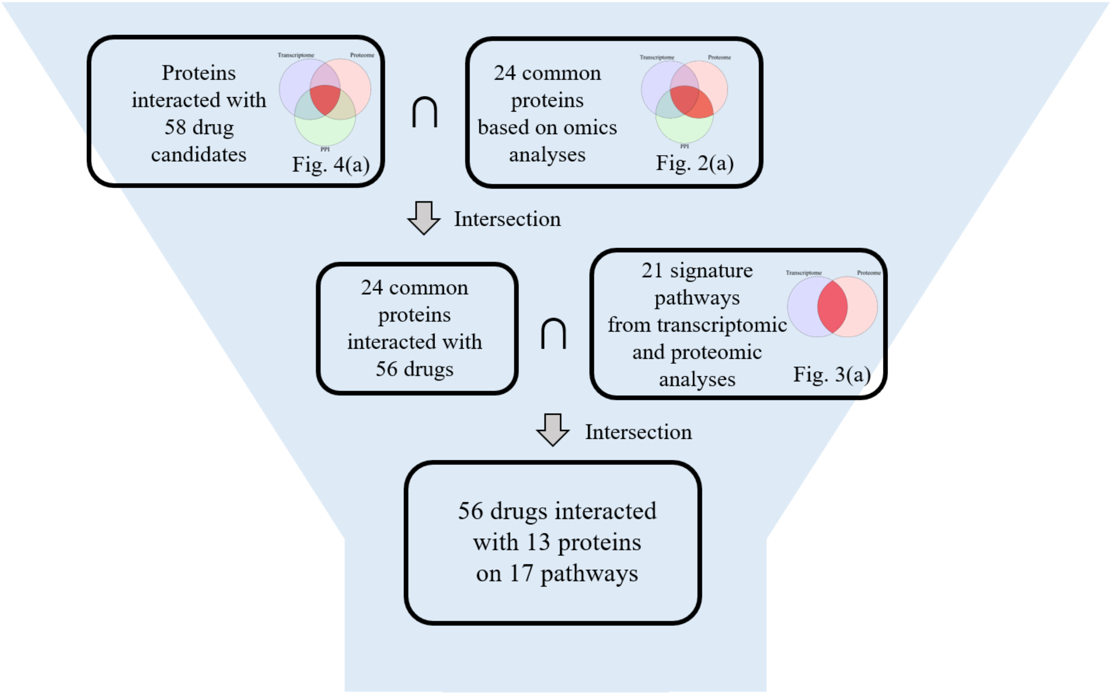

Supplementary Fig. S5. Schematic diagram of the filtering method used to select the drug candidates against DENV infection. Fifty-eight drugs were identified (Fig. 4a). Twenty-four proteins were identified as common signature proteins (Fig. 2a). Twenty-one pathways were identified as signature pathways (Fig. 3a). First, 24 common signature proteins were selected as the product between proteins that interacted with the 58 drugs and 24 common signature proteins. These 24 proteins interacted with 56 of the 58 drugs. Second, 13 proteins were selected as the product between 24 proteins interacting with the 56 drugs and proteins participating in the 21 signature pathways. These 13 proteins interacted with 56 drugs and participated in 17 of the 21 pathways.

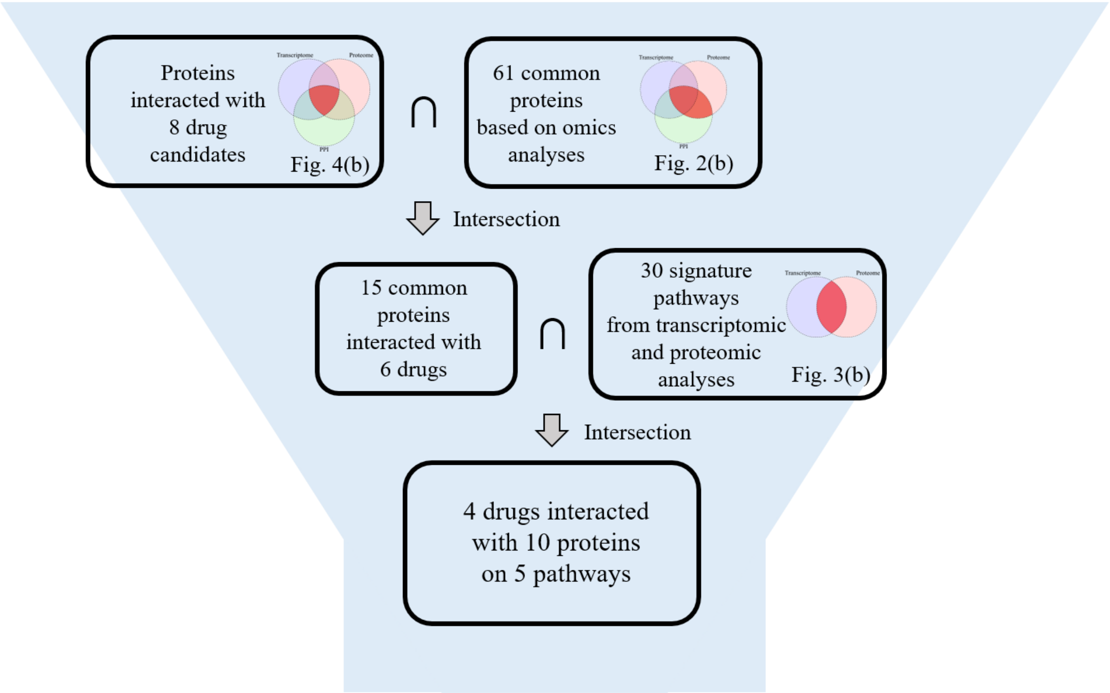

Supplementary Fig. S6. Schematic diagram of the filtering method used to select drug candidates against ZIKV infection. Eight drugs were identified (Fig. 4b). Sixty-one proteins were identified as common signature proteins (Fig. 2b). Thirty pathways were identified as signature pathways (Fig. 3b). First, 15 common signature proteins were selected as the product between proteins that interacted with the eight drugs and 61 common signature proteins. These 15 common signature proteins interacted with six drugs. Second, 10 proteins were selected as the product between the 15 common signature proteins interacting with the six drugs and proteins participating in the 30 signature pathways. These 10 proteins interacted with four drugs and participated in five of the 30 pathways.

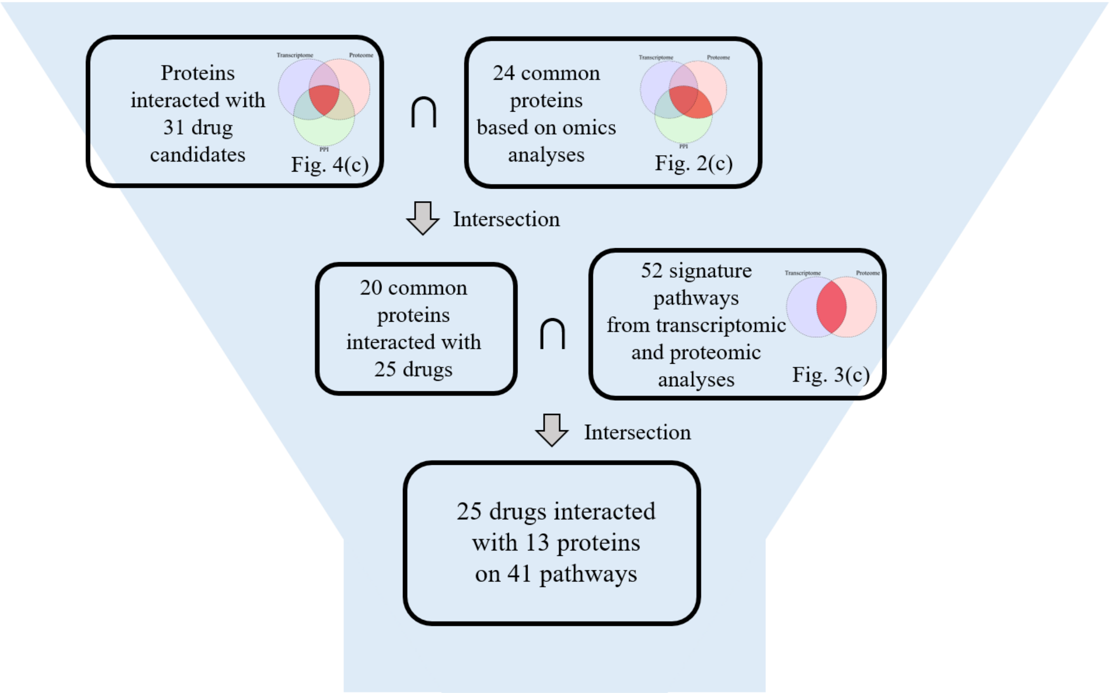

Supplementary Fig. S7. Schematic diagram of the filtering method used to select drug candidates against WNV infection. Thirty-one drugs were identified (Fig. 4c). Twenty-four proteins were identified as common signature proteins (Fig. 2c). Fifty-two pathways were identified as signature pathways (Fig. 3c). First, 20 common signature proteins were selected as the product between proteins that interacted with the 31 drugs and 24 common signature proteins. These 20 proteins interacted with 25 of the 31 drugs. Second, 13 proteins were selected as the product between the 20 common signature proteins interacting with the 25 drugs and proteins participating in the 52 signature pathways. These 13 proteins interacted with 25 drugs and participated in 41 of the 52 pathways.

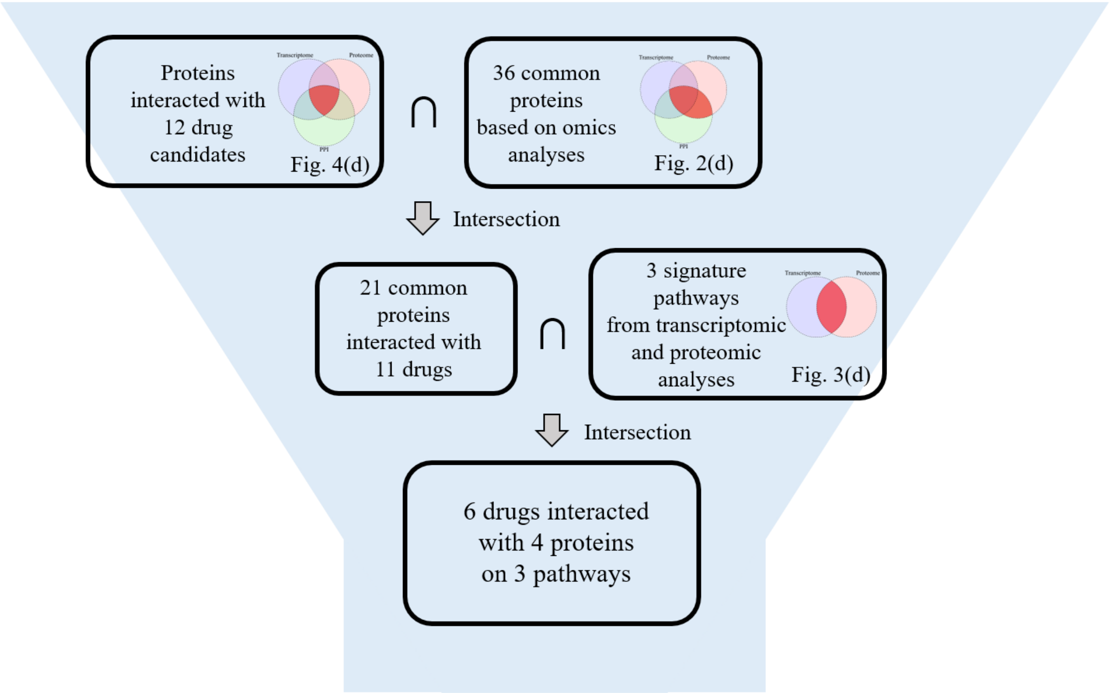

Supplementary Fig. S8. Schematic diagram of the filtering method used to select drug candidates against CHIV infection. Twelve drugs were identified (Fig. 4d). Thirty-six proteins were identified as common signature proteins (Fig. 2d). Three pathways were identified as signature pathways (Fig. 3d). First, 21 common signature proteins were selected as the product between proteins that interacted with the 12 drugs and 36 common signature proteins. These 21 proteins interacted with 11 of the 12 drugs. Second, four proteins were selected as the product between the 21 common signature proteins interacting with the 11 drugs and proteins participating in the three signature pathways. These four proteins interacted with six drugs and participated in all three pathways.

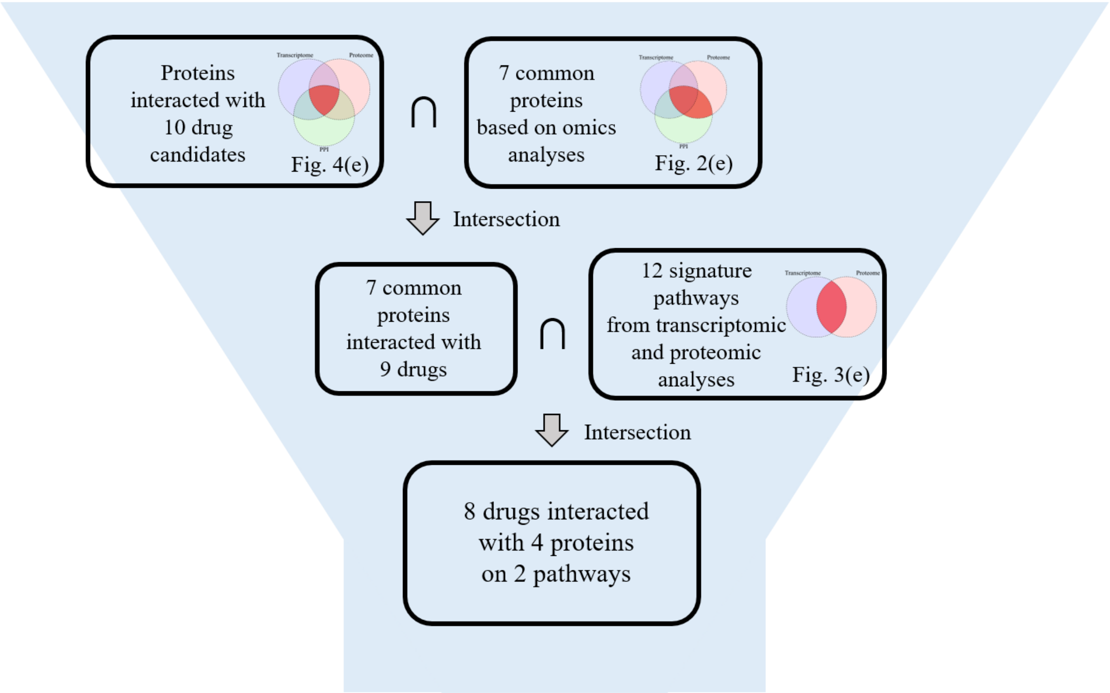

Supplementary Fig. S9. Schematic diagram of the filtering method used to select drug candidates against JEV infection. Ten drugs were identified (Fig. 4e). Seven proteins were identified as common signature proteins (Fig. 2e). Twelve pathways were identified as signature pathways (Fig. 3e). First, seven common signature proteins were selected as the product between proteins that interacted with the 10 drugs and seven common signature proteins. These seven proteins interacted with nine of the 10 drugs. Second, four proteins were selected as the product between the seven common signature proteins interacting with the nine drugs and proteins participating in the 12 signature pathways. These four proteins interacted with eight drugs and participated in two of the 12 pathways.

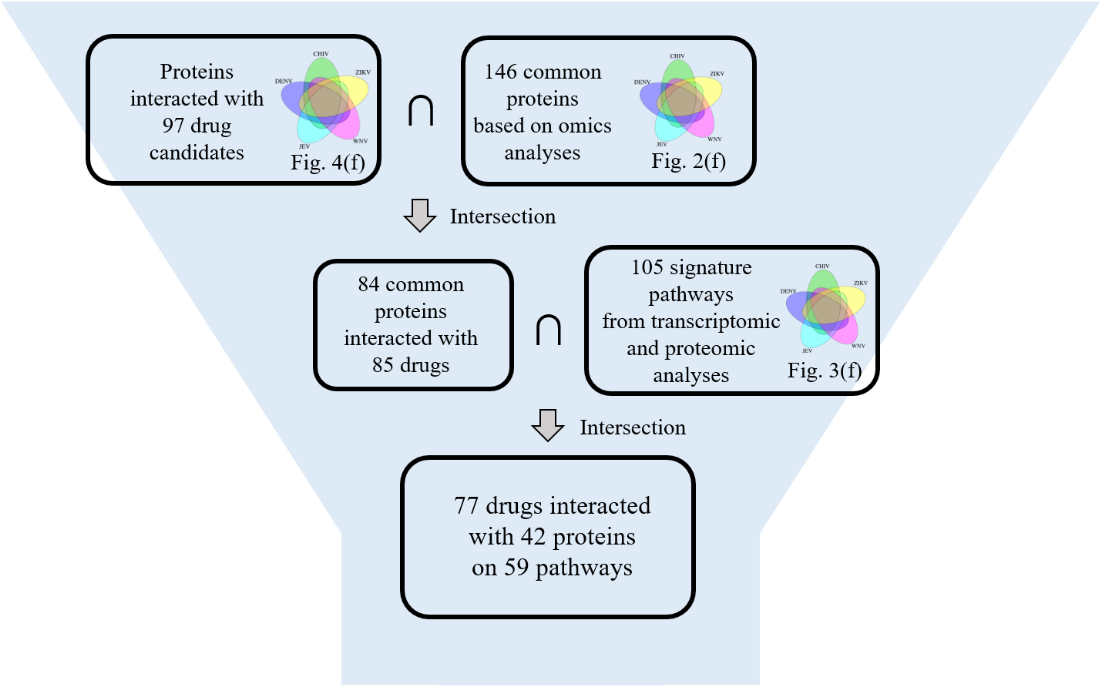

Supplementary Fig. S10. Schematic diagram of the filtering method used to select drug candidates for the five viral infections. A diagram of the process used to filter and select drug candidates based on the common signature proteins (union of common signature proteins in Fig. 2f), signature pathways (union of pathways in Fig. 3f) and drugs (union of drug candidates in Fig. 4f).

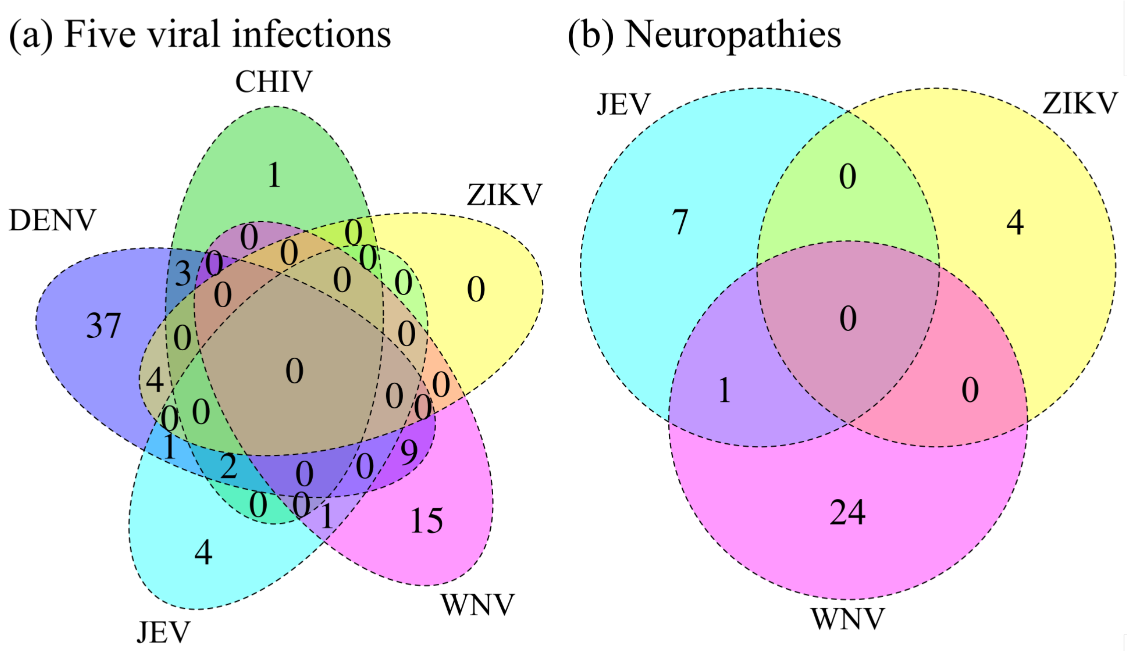

Supplementary Fig. S11. Drug candidates filtered by our methods. The Venn diagrams show the shared number of drug candidates for the five infections (**a**) and neurological complications (**b**).

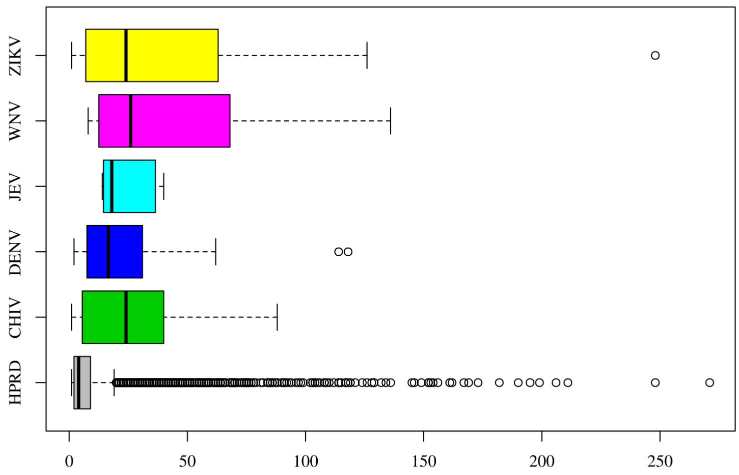

Supplementary Fig. S12. Box plots showing the frequency distribution of the degree of common signature proteins in ZKV (yellow), WNV (pink), JEV (light blue), DENV (blue) and CHIV (green). The box plot for the HPRD (grey) shows the distribution of the degree for all proteins in the PPI network from the HPRD. The average degree of common and extended proteins was 25.59 and 45.64 for DENV, 21.74 and 41.27 for ZIKV, 43.42 and 44.59 for WNV, 21.94 and 49.28 for CHIV, and 24.85 and 45.58 for JEV. The values for the degree of proteins are defined as low (degree ≤ 10), intermediate (10 < degree ≤ 150) and high (degree >150).

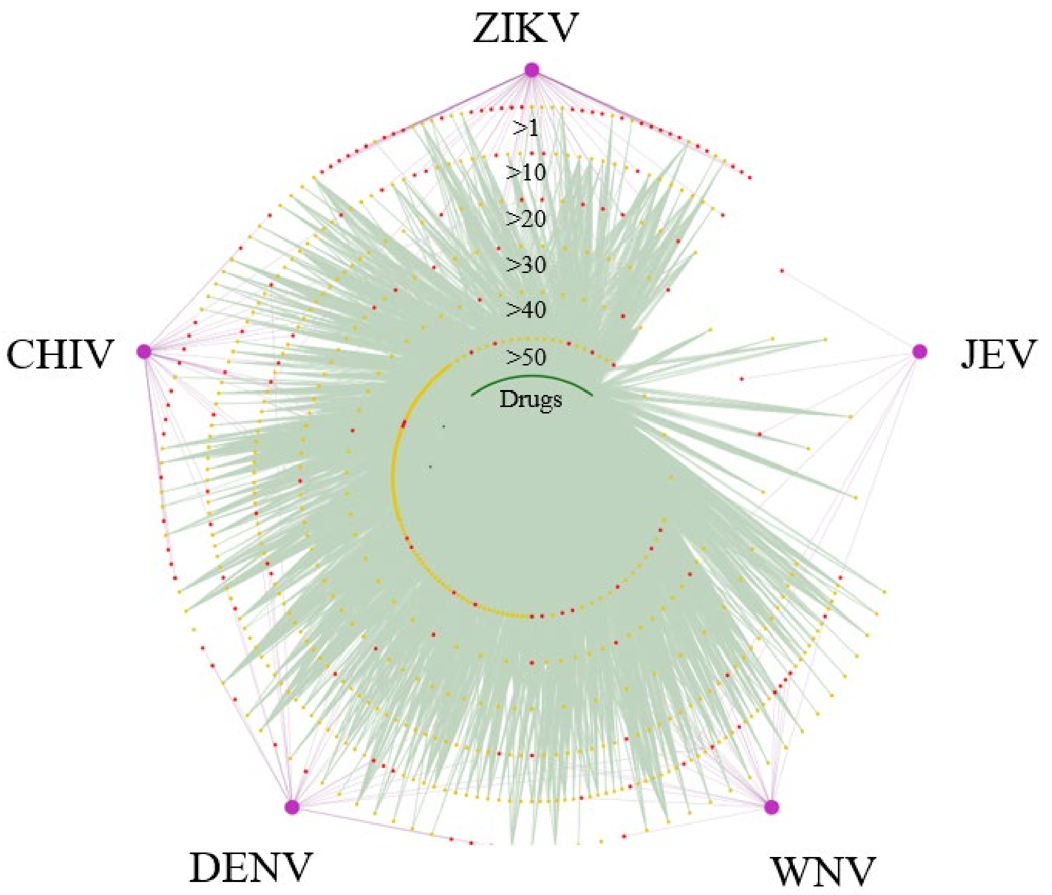

Supplementary Fig. S13. The relationship between drug candidates and proteins. The concentric circles show the network of diseases (purple circles), proteins (red and yellow circles) and drugs (green circles). The degrees of the proteins calculated from the PPI network in the HPRD are divided into 0 to 10, 10 to 20, 20 to 30, 30 to 40, 40 to 50 and 50 or greater. The circularly distributed proteins with smaller radii indicate higher degrees. The numbers of common (red) and extended (yellow) proteins counting from the outer circle inwards are 32, 34, 40, 46, 48 and 57 for common signature proteins and 47, 54, 71, 94, 116 and 190 for extended signature proteins. The purple edge shows the connection between diseases and common signature proteins. The green edge shows the extended signature protein-drug candidate interactions.

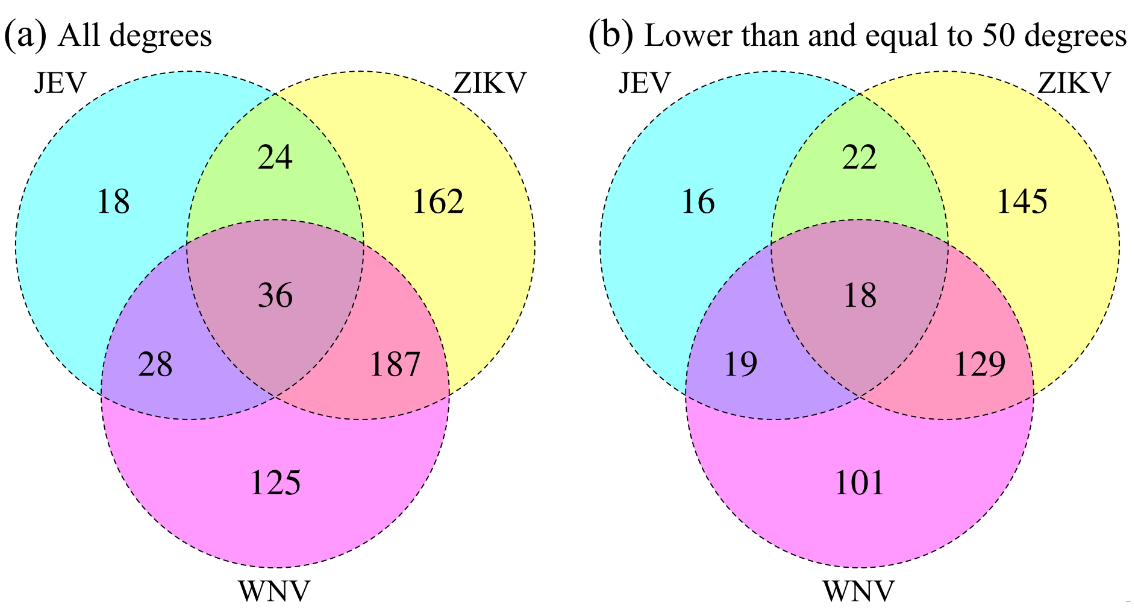

Supplementary Fig. S13. Venn diagrams of the common and extended signature proteins for the neurological complications. Common and extended signature protein numbers with all degrees are shown in (**a**) and those proteins with lower than and equal to 50 degrees are given in (**b**).

Supplementary Table S1. Transcriptomic datasets.

Virus	GSEID	Samples (N: normal, D: disease)	Platform	Infection types
DENV				
	GSE50698	12 (N3,D9)	Affymetrix	Human blood infection
	GSE23986	8 (N4, D4)	Affymetrix	Human blood infection
	GSE51808	56 (N28, D28)	Affymetrix	Patient blood
	GSE34628	6 (N3, D3)	Affymetrix	Infected cell line (HUVEC)
	GSE9378	10 (N5, D5)	Affymetrix	Human blood infection
	GSE18090	26 (N8, D18)	Affymetrix	Patient blood
	GSE40628	34 (N4, D30)	SMD	Patient blood
	GSE38246	113 (N8, D105)	SMD	Patient blood
	GSE25226	44 (N10, D34)	HEEBO	Patient blood
	GSE58278	18 (N6, D12)	Illumina	Infected cell line (CD14)
ZIKV				
	GSE98889	20 (N10, D10)	Affymetrix	Infected cell line (Vero)
WNV				
	GSE46681	195 (N78, D78)	Illumina	Patient blood
	GSE30719	10 (N10, D10)	Affymetrix	Infected cell line (ARPE-19)
CHIV				
	GSE49985	6 (N2, D4)	Affymetrix	Infected cell line (HEK293T)
JEV				
	GSE57330	12 (N6, D6)	Affymetrix	Infected cell line (CHME3)

Ten datasets were collected for DENV. Five datasets were from blood samples of viral infected patients and normal controls as follows: GSE 51808, from 28 patients and 28 normal controls[1]; GSE18090, from 18 patients and eight controls[2]; GSE40528, from 30

patients and four controls[3]; GSE38246, 105 patients and eight controls[4]; and GSE25226, from 34 patients and ten controls[5]. Three datasets were from blood samples of healthy volunteers and cultured cells that were infected with DENV: GSE50698, from three healthy and nine viral infected samples[6]; GSE23986, from four healthy and four viral infected samples[7]; and GSE9378, from three healthy and three viral infected samples[8]. Two datasets were from cell lines and cell lines infected by DENV: GSE34628, from 28 uninfected and 28 infected cell lines (HUVEC)[9] and GSE58278, from 6 healthy and 12 viral infected cell lines (CD14)[10].Two datasets were reported for WNV. One dataset was from 78 blood samples taken from infected patients and 78 controls (GSE46681)[11], and the other dataset was from ten healthy and ten viral infected cell lines (ARPE-19; GSE30719)[12]. One dataset from healthy and viral infected cell lines was reported for ZIKV, CHIV and JEV. The ZIKV dataset was from ten healthy and ten viral infected cell lines (Vero; GSE98889)[13], the CHIV dataset was from two healthy and four viral infected cell lines (HEK 293T; GSE49985)[14], and the JEV dataset was from six healthy and six viral infected cell lines (CHME3; GSE57330)[15].

Supplementary Table S2. Datasets for proteomic analysis.

Virus	PMID	Signature proteins	Up-regulated proteins	Down-regulated proteins
DENV				
	24671231	389	106	285
ZIKV				
	28112162	453	255	198
WNV				
	16192685	9	5	4
	19395707	4	3	1
	26485063	615	540	195
CHIV				
	23593481	45	8	37
	22514668	68	68	0
	24124767	20	10	10
	25764339	384	108	276
	26083627	20	5	15
JEV				
	27563890	28	17	11
	23647205	155	56	99

Chiu et al.[16] reported a proteomic analysis of human A549 cells in response to infection with DENV type 2 using high-throughput mass spectrometry and identified ~4,000 proteins with significant differences in abundances between DENV infected and normal cells. The results of this study also showed that 94.5% and 90% of proteins in the cytoplasmic and nuclear fractions, respectively, showed a >1.5-fold change in abundance following DENV type 2 infection. Garcez et al.[17] detected 199 downregulated and 259 upregulated proteins when comparing the proteomes of ZIKV-infected to mock-infected neurospheres. Dhingra et al.[18] identified 93 animal-derived proteins, including three human proteins, using proteomics technology to analyze early Vero cells response to WNV infection. Pastorino et al.[19] examined a WNV-infected neuron culture by high-resolution two-dimensional fluorescence difference gel electrophoresis (2D-DIGE) and identified four proteins that were downregulated and five proteins that were upregulated when compared with uninfected cells as the control. Abere et al.[20] used GeLC-MS/MS analysis to show that 90 proteins were downregulated significantly in CHIKV infected CHME-5 cells when compared with that of mock infected CHME-5 cells. Thio et al.[21] identified 53 proteins whose

expressions were different when comparing whole cell proteome profiles of CHIV-infected and mock control WRL-68 cells by two-dimensional gel electrophoresis (2-DGE). Puttamallesh et al.[22] found 63 proteins by proteomic profiling of sera to be differentially expressed in CHIV infected patients when compared with that of control patients. Treffers et al.[23] showed by SILAC and LC-MS/MS analysis that at 8, 10 and 12 h post infection in 293/ACE2 cells, 13, 38 and 106 proteins, respectively, were differentially expressed. Thio et al.[24] identified 25 regulated proteins in CHIKV infected cells. Zhang et al.[25] identified by a SILAC-based quantitative proteomics approach 158 host proteins as differentially regulated by JEV for JEV-infected HeLa cells. In each infection, signature proteins were identified and protein expression data are presented.

Supplementary Table S3. Datasets for interactomic analyses.

Virus	PMID	Viral-human PPIs	Human proteins
DENV			
	29049286	525	342
	21281507	36	31
	21911577	139	105
	22014111	46	45
	23326450	52	47
ZIKV			
	30177828	560	499
	29197720	27	23
WNV			
	20308361	1	1
	21642539	1	1
	19712667	1	1
	19889084	1	1
	20417716	3	3
	23522008	1	1
	18061925	1	1
	21767858	2	2
	11920827	2	1
	12966555	2	1
	15475343	1	1
	17132743	1	1
	16415006	2	1
	19635919	1	1
	17868381	1	1
	23876037	1	1
	19846531	1	1
	22014111	6	6
	16275649	1	1
	18005741	1	1
	24478428	2	2
	16854374	2	2
	16882664	2	2

	19451286	1	1
	21411523	1	1
	23137297	1	1
CHIV			
	25782748	30	30
	22258240	103	75
	24462973	1	1
	25563600	55	53
	26384002	1	1
	23334837	94	75
	26829480	1	1
	22951312	10	6
	12584323	1	1
JEV			
	21767858	1	1
	26819305	2	1
	19068261	1	1
	28077444	4	4
	21281954	2	2
	21865391	3	2
	23097442	2	2
	24035833	6	2
	24418539	2	1
	18588927	1	1
	28468311	1	1
	29593046	1	1
	26629950	1	1
	30082709	1	1
	28053106	2	2
	19451286	1	1
	24009510	1	1
	16547420	1	1
	21999493	1	1
	24086464	4	3
	28179530	1	1
	21940409	1	1

The literature search found five studies that reported experimental evidence for interactions between human proteins and DENV proteins based on high-throughput yeast two-hybrid screening methods. Khadka et al.[26] reported 139 interactions between DENV and human proteins, and the majority of these interactions were novel. Le Breton et al.[27] focused on the NS3 and NS5 viral proteins, which are major enzymatic components of the viral replication complex and essential to the flavivirus life cycle. This study reported 186 interactions between DENV and human proteins with 171 of the interactions identified by yeast two-hybrid methods and 16 taken from published data. Mairiang et al.[28] identified 46 interactions, including six that had been reported previously. Recently, Dey and Mukhopadhyay[29] reported the development of DenvInt, which is a database of manually curated experimental data of DENV and host protein interactions. Two studies reported experimental evidence for interactions between human proteins and ZIKV proteins[30,31]. Twenty-six studies showed experimental evidence for interactions between human proteins and WNV proteins. Nine studies reported experimental evidence for interactions between human proteins and CHIV proteins, and 21 studies provided experimental data for interactions between human and JEV proteins.

Supplementary Table S4. Drug candidates filtered in our multi-omics analyses.

References

1 Kwissa, M. *et al.* Dengue virus infection induces expansion of a CD14(+)CD16(+) monocyte population that stimulates plasmablast differentiation. *Cell Host Microbe* **16**, 115-127, doi:10.1016/j.chom.2014.06.001 (2014).

2 Nascimento, E. J. *et al.* Gene expression profiling during early acute febrile stage of dengue infection can predict the disease outcome. *PLoS One* **4**, e7892, doi:10.1371/journal.pone.0007892 (2009).

3 Khadem, A. F., Pol, A., Wieczorek, A. S., Jetten, M. S. & Op den Camp, H. J. Metabolic Regulation of "Ca. Methylacidiphilum Fumariolicum" SolV Cells Grown Under Different Nitrogen and Oxygen Limitations. *Front Microbiol* **3**, 266, doi:10.3389/fmicb.2012.00266 (2012).

4 Popper, S. J. *et al.* Temporal dynamics of the transcriptional response to dengue virus infection in Nicaraguan children. *PLoS Negl Trop Dis* **6**, e1966, doi:10.1371/journal.pntd.0001966 (2012).

5 Loke, P. *et al.* Gene expression patterns of dengue virus-infected children from nicaragua reveal a distinct signature of increased metabolism. *PLoS Negl Trop Dis* **4**, e710, doi:10.1371/journal.pntd.0000710 (2010).

6 Silveira, G. F. *et al.* Single point mutations in the helicase domain of the NS3 protein enhance dengue virus replicative capacity in human monocyte-derived dendritic cells and circumvent the type I interferon response. *Clin Exp Immunol* **183**, 114-128, doi:10.1111/cei.12701 (2016).

7 Silveira, G. F. *et al.* Dengue virus type 3 isolated from a fatal case with visceral complications induces enhanced proinflammatory responses and apoptosis of human dendritic cells. *J Virol* **85**, 5374-5383, doi:10.1128/JVI.01915-10 (2011).

8 Warke, R. V. *et al.* TRAIL is a novel antiviral protein against dengue virus. *J Virol* **82**, 555-564, doi:10.1128/JVI.01694-06 (2008).

9 Dalrymple, N. A. & Mackow, E. R. Endothelial cells elicit immune-enhancing responses to dengue virus infection. *J Virol* **86**, 6408-6415, doi:10.1128/JVI.00213-12 (2012).

10 Olagnier, D. *et al.* Cellular oxidative stress response controls the antiviral and apoptotic programs in dengue virus-infected dendritic cells. *PLoS Pathog* **10**, e1004566, doi:10.1371/journal.ppat.1004566 (2014).

11 Qian, F. *et al.* Systems immunology reveals markers of susceptibility to West Nile virus infection. *Clin Vaccine Immunol* **22**, 6-16, doi:10.1128/CVI.00508-14 (2015).

12 Munoz-Erazo, L., Natoli, R., Provis, J. M., Madigan, M. C. & King, N. J. Microarray analysis of gene expression in West Nile virus-infected human retinal pigment epithelium. *Mol Vis* **18**, 730-743 (2012).

13 Mladinich, M. C., Schwedes, J. & Mackow, E. R. Zika Virus Persistently Infects and Is Basolaterally Released from Primary Human Brain Microvascular Endothelial Cells. *MBio* **8**, doi:10.1128/mBio.00952-17 (2017).

14 Saxena, T. *et al.* Combined miRNA and mRNA signature identifies key molecular players and pathways involved in chikungunya virus infection in human cells. *PLoS One* **8**, e79886, doi:10.1371/journal.pone.0079886 (2013).

15 Kumari, B. *et al.* Dynamic changes in global microRNAome and transcriptome reveal complex miRNA-mRNA regulated host response to Japanese Encephalitis Virus in microglial cells. *Sci Rep* **6**, 20263, doi:10.1038/srep20263 (2016).

16 Chiu, H. C., Hannemann, H., Heesom, K. J., Matthews, D. A. & Davidson, A. D. High-throughput quantitative proteomic analysis of dengue virus type 2 infected A549 cells. *PLoS One* **9**, e93305, doi:10.1371/journal.pone.0093305 (2014).

17 Garcez, P. P. *et al.* Zika virus disrupts molecular fingerprinting of human neurospheres. *Sci Rep* **7**, 40780, doi:10.1038/srep40780 (2017).

18 Dhingra, V., Li, Q., Allison, A. B., Stallknecht, D. E. & Fu, Z. F. Proteomic profiling and neurodegeneration in West-Nile-virus-infected neurons. *J Biomed Biotechnol* **2005**, 271-279, doi:10.1155/JBB.2005.271 (2005).

19 Pastorino, B. *et al.* Identification of cellular proteome modifications in response to West Nile virus infection. *Mol Cell Proteomics* **8**, 1623-1637, doi:10.1074/mcp.M800565-MCP200 (2009).

20 Abere, B. *et al.* Proteomic analysis of chikungunya virus infected microgial cells. *PLoS One* **7**, e34800, doi:10.1371/journal.pone.0034800 (2012).

21 Thio, C. L., Yusof, R., Abdul-Rahman, P. S. & Karsani, S. A. Differential proteome analysis of chikungunya virus infection on host cells. *PLoS One* **8**, e61444, doi:10.1371/journal.pone.0061444 (2013).

22 Puttamallesh, V. N. *et al.* Proteomic profiling of serum samples from chikungunya-infected patients provides insights into host response. *Clin Proteomics* **10**, 14, doi:10.1186/1559-0275-10-14 (2013).

23 Treffers, E. E. *et al.* Temporal SILAC-based quantitative proteomics identifies host factors involved in chikungunya virus replication. *Proteomics* **15**, 2267-2280, doi:10.1002/pmic.201400581 (2015).

24 Thio, C. L. *et al.* Differential Analysis of the Secretome of WRL68 Cells Infected with the Chikungunya Virus. *PLoS One* **10**, e0129033, doi:10.1371/journal.pone.0129033 (2015).

25 Zhang, L. K., Chai, F., Li, H. Y., Xiao, G. & Guo, L. Identification of host proteins involved in Japanese encephalitis virus infection by quantitative proteomics analysis. *J*

Proteome Res **12**, 2666-2678, doi:10.1021/pr400011k (2013).

26 Khadka, S. *et al.* A physical interaction network of dengue virus and human proteins. *Mol Cell Proteomics* **10**, M111 012187, doi:10.1074/mcp.M111.012187 (2011).

27 Le Breton, M. *et al.* Flavivirus NS3 and NS5 proteins interaction network: a high-throughput yeast two-hybrid screen. *BMC Microbiol* **11**, 234, doi:10.1186/1471-2180-11-234 (2011).

28 Mairiang, D. *et al.* Identification of new protein interactions between dengue fever virus and its hosts, human and mosquito. *PLoS One* **8**, e53535, doi:10.1371/journal.pone.0053535 (2013).

29 Dey, L. & Mukhopadhyay, A. DenvInt: A database of protein-protein interactions between dengue virus and its hosts. *PLoS Negl Trop Dis* **11**, e0005879, doi:10.1371/journal.pntd.0005879 (2017).

30 Scaturro, P. *et al.* An orthogonal proteomic survey uncovers novel Zika virus host factors. *Nature* **561**, 253-257, doi:10.1038/s41586-018-0484-5 (2018).

31 Gurumayum, S. *et al.* ZikaBase: An integrated ZIKV- Human Interactome Map database. *Virology* **514**, 203-210, doi:10.1016/j.virol.2017.11.007 (2018).
